# Cerebellar Purkinje cells can differentially modulate coherence between sensory and motor cortex depending on region and behavior

**DOI:** 10.1073/pnas.2015292118

**Published:** 2020-12-22

**Authors:** Sander Lindeman, Sungho Hong, Lieke Kros, Jorge F. Mejias, Vincenzo Romano, Robert Oostenveld, Mario Negrello, Laurens W. J. Bosman, Chris I. De Zeeuw

**Affiliations:** ^a^Department of Neuroscience, Erasmus MC, 3015 GE Rotterdam, The Netherlands;; ^b^Sensory and Behavioural Neuroscience Unit, Okinawa Institute of Science and Technology, 904-0495 Okinawa, Japan;; ^c^Computational Neuroscience Unit, Okinawa Institute of Science and Technology, 904-0495 Okinawa, Japan;; ^d^Swammerdam Institute for Life Sciences, University of Amsterdam, 1098 XH Amsterdam, The Netherlands;; ^e^Donders Institute for Brain, Cognition and Behaviour, Radboud University, 6500 GL Nijmegen, The Netherlands;; ^f^The Swedish National Facility for Magnetoencephalography (NatMEG), Karolinska Institutet, 171 65 Solna, Sweden;; ^g^Netherlands Institute for Neuroscience, Royal Academy of Arts and Sciences, 1105 BA Amsterdam, The Netherlands

**Keywords:** cerebellum, cerebral cortex, whisker system, laminar model, LFP

## Abstract

Activity of sensory and motor cortices is essential for sensorimotor integration. In particular, coherence between these areas may indicate binding of critical functions like perception, motor planning, action, or sleep. Evidence is accumulating that cerebellar output modulates cortical activity and coherence, but how, when, and where it does so is unclear. We studied activity in and coherence between S1 and M1 cortices during whisker stimulation in the absence and presence of optogenetic Purkinje cell stimulation in crus 1 and 2 of awake mice, eliciting strong simple spike rate modulation. Without Purkinje cell stimulation, whisker stimulation triggers fast responses in S1 and M1 involving transient coherence in a broad spectrum. Simultaneous stimulation of Purkinje cells and whiskers affects amplitude and kinetics of sensory responses in S1 and M1 and alters the estimated S1–M1 coherence in theta and gamma bands, allowing bidirectional control dependent on behavioral context. These effects are absent when Purkinje cell activation is delayed by 20 ms. Focal stimulation of Purkinje cells revealed site specificity, with cells in medial crus 2 showing the most prominent and selective impact on estimated coherence, i.e., a strong suppression in the gamma but not the theta band. Granger causality analyses and computational modeling of the involved networks suggest that Purkinje cells control S1–M1 phase consistency predominantly via ventrolateral thalamus and M1. Our results indicate that activity of sensorimotor cortices can be dynamically and functionally modulated by specific cerebellar inputs, highlighting a widespread role of the cerebellum in coordinating sensorimotor behavior.

## Significance

Coordinated activity of sensory and motor cortices is essential for adjusting movements based on sensory feedback. Sensory and motor cortices communicate directly as well as via the thalamus and also receive indirect input from the cerebellum. We show here that cerebellar activity can affect the amplitude and coherence of fast sensorimotor responses in the primary somatosensory and motor cortices upon whisker stimulation. The cerebellum can differentially alter sensory-induced theta- and gamma-band cortical coherences via a fast ascending pathway. In line with the functional heterogeneity of its modular organization, cerebellar impact is region-specific and tuned to ongoing motor responses. Our data highlight site-specific and context-dependent cerebello-cerebral interactions that can come into play during a plethora of sensorimotor functions.

Integration of sensory feedback into motor control is of obvious importance for the learning and execution of skilled movements. This integration is particularly relevant when subjects explore their environment via active touch, requiring sensory input to be directly related to the momentary position and movement of eyes, fingertips, antennae, whiskers, or other sense organs (1–3). In vertebrates, adapting movement based upon somatosensory feedback requires collaborative action of primary somatosensory (S1) and motor (M1) cortices (4–6), which are directly and reciprocally connected (7–12). The reciprocal connections between S1 and M1 imply that each region can differentially affect the amplitude of neuronal responses in the other region. These connections can also create coherent activity patterns in S1 and M1 (13–17).

Coherence can add an extra layer of neuronal integration, as it has been suggested to bind different brain areas by affecting susceptibility of neurons to synaptic input and providing a timing mechanism for generating a common dynamical frame for cortical operations (18–22). As such, coherence can create a temporal framework for concerted neural activity that facilitates integration of the activity of sensory and motor areas (23–25). Coherence often occurs in specific frequency bands that can be associated with different functions. In the field of sensorimotor integration, skilled movements rely on intercortical coherence between sensory and motor areas that occur in the theta range (4 to 8 Hz) during force generation, while coherence at higher bands is engaged during the preparation thereof (26). Likewise, within the field of visual perception, coherence in the alpha (8 to 12 Hz) and gamma (30 to 100 Hz) bands have been found to contribute to feedback and feedforward processing, respectively (27, 28).

Cortical coherence can be modulated by subcortical activity, as has been particularly well established for the thalamus (29–32). Accordingly, the cerebellum, one of the main inputs to the thalamus, has a strong impact in organizing cortical coherence (33–35). Indeed, disruption of cerebellar function, whether inflicted pharmacologically in rats (34) or by stroke in patients (33), affects cortico-cortical coherence.

Given the strong cerebello-thalamo-cortical projections (36, 37), it is not surprising that cerebellar activity affects the amplitude and coherence of S1 and M1 (20, 34, 38, 39). However, it is unclear to what extent cerebellar activity can modulate the kinetics of the fast sensory responses in S1 that are propagated by the canonical lemniscal and paralemniscal pathways (40), whether it can differentially influence individual frequency bands, to what extent such potentially different impacts depend on the behavioral context, and whether these differential effects are mediated through different cerebellar modules (41–43). Here, we set out to address these questions by investigating the impact of Purkinje cell activity on the response amplitude and the estimated coherence between the whisker areas of the primary somatosensory (wS1) and motor cortex (wM1) during stimulation of the whiskers in awake behaving mice. Stimulating the whiskers results in transient sensory responses in wS1 and wM1, which were affected by optogenetic stimulation of Purkinje cells at different intervals with respect to air-puff stimulation of the whiskers. The estimated coherence of the sensory response in wS1 and wM1 was differentially modulated by Purkinje cell stimulation, affecting mainly the activity in the theta and gamma bands, depending on ongoing behavior and their precise site in the cerebellar hemispheres.

## Results

### Sensory Responses in wS1 Are Affected by Purkinje Cell Stimulation.

**Fig. 1.**
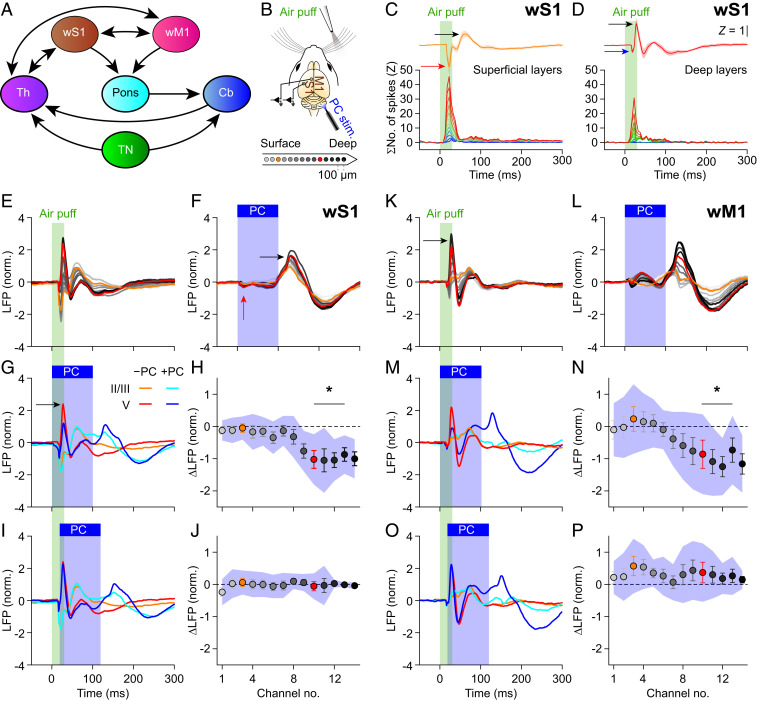
Purkinje cell stimulation disinhibits fast sensory responses in wS1 and wM1. (*A*) Highly simplified anatomical scheme. *SI Appendix*, Fig. S1*A*, shows a more realistic scheme. Cb, cerebellum; Th, thalamus; TN, trigeminal nuclei. (*B*) LFPs were recorded in wS1 and wM1 of awake mice using, for each area, 14 recording spots on linear silicon probes. Colors indicate their relative positions, with orange and red for the 3rd and 10th electrodes, representing the supra- and subgranular layers, respectively. (*C* and *D*) Air-puff stimulation triggered a response in superficial (*C*) and deeper layers (*D*) of wS1. Averaged multiunit activity of 12 and 10 recording sites, respectively, showing that the peak in spike response corresponded to the LFP trough [averaged *Z*-scored LFP traces of electrodes 2 to 6 (*C*) and 7 to 14 (*D*) of 8 mice; *SI Appendix*, Fig. S2 *C*–*F*]. (*E*) Whisker stimulation triggered fast responses in the LFP of contralateral wS1 (first, per mouse, 100 trials per condition were averaged, then an average of 8 mice was made). Color gradient as in *B*. (*F*) Purkinje cells (PCs) were stimulated optogenetically using an optic fiber with 400 µm diameter placed on the center of crus 1 (*SI Appendix*, Fig. S2*G*). This triggered delayed responses in wS1 after rebound firing in the cerebellar nuclei (*SI Appendix*, Fig. S3*B*). (*G*) Comparison of the LFPs recorded during trials with whisker stimulation (orange/red) and with combined sensory and Purkinje cell stimulation (cyan/blue). (*H*) During the early response period, the amplitude of the first positive LFP peak in the subgranular layers was especially affected, in addition to profound impact during later phases of sensory processing. Plotted are the averaged differences in amplitude of the first positive LFP peaks. Error bars indicate SEM and shaded area SD. (*I* and *J*) The impact of optogenetic PC stimulation on the first positive LFP peak was largely abolished by introducing a 20-ms delay between the start of air-puff sensory stimulation and the onset of optogenetic PC stimulation. (*K***–***P*) The same plots as in *E* to *J*, but for wM1, showing comparable impact of optogenetic PC stimulation on the sensory-induced LFP signals.

Sensory input from the facial whiskers reaches wS1 via strong, well-characterized trigemino-thalamo-cortical pathways, resulting in fast sensory responses in wS1 (3, 40, 44, 45) (Fig. 1*A* and *SI Appendix*, Fig. S1*A*). Accordingly, we observed a rapid increase in multiunit activity in wS1 upon air-puff stimulation of the contralateral whiskers. This increased spiking activity was associated with a negative peak in the intracortical local field potential (LFP) signal (Fig. 1 *B*–*D* and *SI Appendix*, Figs. S1 *B* and *C* and S2 *A*–*E*), which is in line with previous experimental and theoretical studies on the relation between spiking activity and LFP signals (46, 47). The positive LFP peak was not unequivocally related to changes in spiking rate, and could be more related to active membrane currents and possibly also to synaptic currents (48).

Whisker stimulation triggered a stronger response in the superficial layers of wS1, as evidenced by the combination of a stronger peak in multiunit activity and a larger negative deflection of the LFP signal, than it did in the deep layers (Fig. 1 *C* and *D*, red and blue arrows; see also *SI Appendix*, Fig. S2*E*). The initial fast decrease in the LFP signal was followed by an increase (Fig. 1 *C* and *D*, black arrows) and finally a return to baseline after roughly 200 ms (Fig. 1*E* and *SI Appendix*, Fig. S3*A*). Despite some interexperimental variations, this pattern was observed in all eight mice studied under these conditions (*SI Appendix*, Fig. S4).

Using current source density analysis, we visualized the sources (red/yellow) and sinks (blue/white) of ionic currents across the layers of wS1. This revealed that the excitation first arrived in layer IV (*SI Appendix*, Fig. S5*A*, yellow arrow), which is the main target of the lemniscal pathway (3, 40), and subsequently spread to deeper as well as to more superficial layers, as pointed out by the magenta arrows in *SI Appendix*, Fig. S5*A*.

Given the importance of the cerebellum for sensorimotor integration (39, 41), we were interested in the putative impact of cerebellar activity on the sensory representation in wS1. As schematized in *SI Appendix*, Fig. S1*A*, the cerebellum receives whisker sensory input from the trigeminal nuclei via direct and indirect pathways (3, 49, 50), resulting in fast sensory responses in multiple regions of the cerebellar cortex, but especially in lobules crus 1 and crus 2 (43, 51–55). In order to test the impact of cerebellar activity on the responses in wS1 to whisker stimulation, we randomly intermingled trials with whisker stimulation, optogenetic stimulation of cerebellar Purkinje cells, and combinations of both stimuli. As Purkinje cells are inhibitory neurons (56), their optogenetic stimulation resulted in a near-complete block of neural activity in a part of the cerebellar nuclei, strongly reducing a part of the cerebellar output for around 100 ms (*SI Appendix*, Fig. S3*B*; see also ref. 57).

Given the strength of the direct trigemino-thalamo-cortical pathways (3, 40), a substantial impact of optogenetic Purkinje cell stimulation on the first phase of the response in wS1 would not be expected. Indeed, neither the fast sensory-induced increase in neuronal spiking in wS1 (*P* = 0.463, W = 47, *n* = 22, Wilcoxon matched-pairs test; *SI Appendix*, Fig. S2 *E*–*G*) nor the related initial negative LFP peak in the input layer was affected by simultaneous optogenetic stimulation of Purkinje cells in crus 1 (*SI Appendix*, Fig. S6 *A* and *B*, red arrow; Table S1 provides statistical details). Simultaneous stimulation of Purkinje cells did reduce, however, the subsequent positive LFP peak in the deeper layers (*P* = 0.024; Fig. 1 *G* and *H* and *SI Appendix*, Fig. S3*A*; see Fig. 1*G*, black arrow, and *SI Appendix*, Fig. S5*B*, white arrow). Thus, while the first phase of the sensory response in wS1 could not be modulated by simultaneous Purkinje cell stimulation, immediately afterward, an impact of cerebellar activity was found. In contrast to the LFP, the multiunit activity in wS1 was not consistently different between trials with and without optogenetic Purkinje cell stimulation (*SI Appendix*, Fig. S2*E*), highlighting that subthreshold activity presumably also contributes to LFP signals (47, 48).

We wondered whether the impact of Purkinje cell stimulation on sensory responses in wS1 was indeed due to a fast pathway via the cerebellum. To this end, we analyzed the trials in which the optogenetic stimulation had been delayed by 20 ms relative to the start of the air puff. In these trials, a fast sensory response in the cerebellum could take place despite the optogenetic stimulation applied immediately afterward (Fig. 1 *I* and *J* and *SI Appendix*, Figs. S3*A* and S5*C* and Table S1). Thus, the introduction of a short delay of the optogenetic stimulation of Purkinje cells directly after the start of sensory stimulation was sufficient to largely restore the normal signal transduction in wS1.

When we stimulated, as a control, the Purkinje cells in the absence of sensory stimulation, we observed a small decrease of the LFP signal in wS1 shortly after stimulation onset, followed by a significantly increased LFP peak after stimulus offset (Fig. 1*F*, red and black arrows, respectively). This sequence of events most likely reflects a near-complete block of the output of the downstream cerebellar nuclei neurons, followed by rebound firing at the end of the 100-ms stimulus interval (*SI Appendix*, Fig. S3*B*; see also ref. 57).

The event-related LFP responses revealed differential impact of cerebellar activation on fast and slow components (Fig. 1 *E*–*G* and *SI Appendix*, Fig. S3*A*). We therefore also examined the spectral properties of the LFP responses. In comparison to whisker stimulation alone, simultaneous whisker and Purkinje cell stimulation induced much less activity in the gamma range (but also in the lower-frequency bands), with this effect being larger in the deeper than in the superficial layers of wS1 (*SI Appendix*, Fig. S7*B*, white arrow; see also *SI Appendix*, Fig. S8). Here too, the impact of Purkinje cell stimulation on sensory-induced oscillatory activity was reduced by a delay of 20 ms (*SI Appendix*, Figs. S7 *A* and *B* and S8).

### Purkinje Cell Stimulation also Affects Sensory Responses in wM1.

Like in wS1, air-puff stimulation to the whiskers also evoked fast responses in wM1. However, while the first phase of the sensory response of wS1 was not affected by simultaneous Purkinje cell stimulation, we found that, in wM1, simultaneous Purkinje cell stimulation could suppress the first phase of sensory-evoked multiunit activity (*P* < 0.001, *F* = 21.385, df = 2, Friedman’s two-way ANOVA; *SI Appendix*, Fig. S2*E*, black arrow). Yet here too, the multiunit response could be rescued by delaying Purkinje cell stimulation 20 ms relative to whisker stimulation (compared to air-puff–only stimulation, *P* = 0.170, *F =* 0.538; *SI Appendix*, Fig. S2 *E*–*G*). Likewise, the first positive peak of the LFP signals in wM1 was affected (*P* = 0.024) by simultaneous (Fig. 1 *K*–*N* and *SI Appendix*, Figs. S3*A*, S4, and S5 *D* and *E*), but not by delayed (Fig. 1 *O* and *P* and *SI Appendix*, Fig. S5*F*), optogenetic stimulation. Thus, our data indicate a fast ascending pathway via the cerebellum affecting the amplitude of neural responses to somatosensory input not only in wS1, but also in wM1.

Like in wS1, Purkinje cell stimulation differentially affected fast and slow components of the sensory responses in wM1, with—as in wS1—a particularly obvious reduction in gamma-band activity after simultaneous sensory and Purkinje cell stimulation (*SI Appendix*, Fig. S7*C*, white arrow). Given that the strongest anatomical connections between wS1 and wM1 are observed between their respective deep and superficial layers (11, 12, 25) (*SI Appendix*, Fig. S1*A*), we compared the phases of sensory responses between deep wS1 and superficial wM1. Doing so, we noticed that wS1 led wM1 at most frequencies, except in the higher gamma band (*SI Appendix*, Fig. S9). During optogenetic stimulation of Purkinje cells alone, there was relatively little phase difference in the gamma-band range, but wM1 led wS1 in the lower bands (delta, theta, and alpha; *SI Appendix*, Fig. S9*B*). During the combined stimulus, wM1 led in the delta and theta bands and wS1 led in the alpha, beta, and lower gamma bands (*SI Appendix*, Fig. S9). These results suggest that wS1 is leading in most of the sensory responses, while wM1 leads the lower frequencies during Purkinje cell stimulation.

### Cerebellum Differentially Modulates Estimated S1–M1 Coherence in Theta and Gamma Bands.

**Fig. 2.**
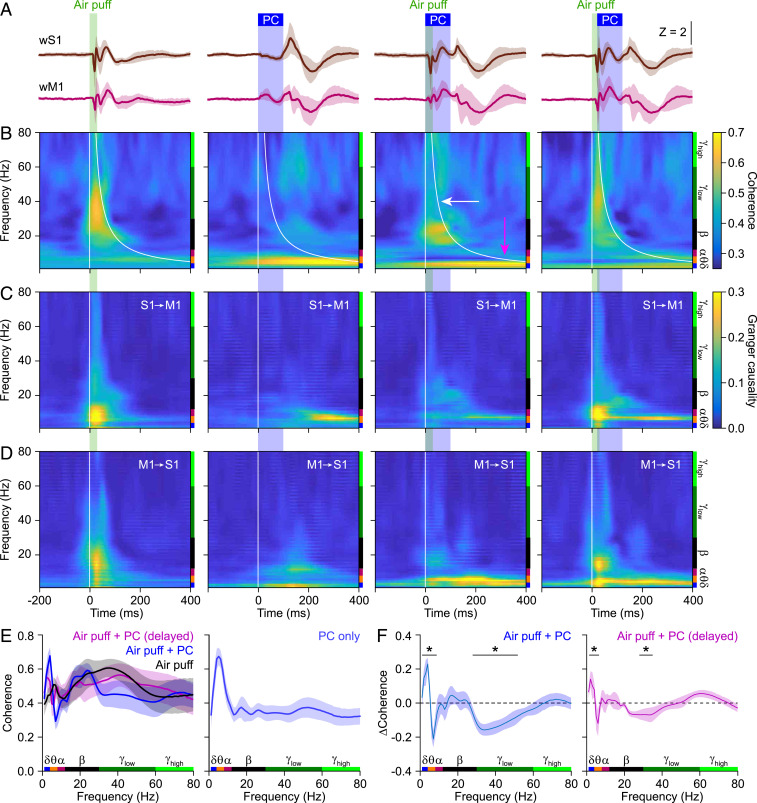
Reducing cerebellar output enhances and inhibits the estimated sensory-induced S1–M1 theta- and gamma-band coherence, respectively. (*A*) Averaged LFP signals in subgranular wS1 and supragranular wM1 following either air-puff stimulation of the contralateral facial whiskers, optogenetic Purkinje cell (PC) stimulation, or a combination of both. In the column on the right, there was a 20-ms delay between the onset of air puff and Purkinje cell stimulation. Purkinje cell stimulation was performed with an optic fiber with a 400-µm diameter placed on the center of crus 1. Traces were derived from channels 10 (wS1) and 3 (wM1), first averaged per mouse (100 trials per condition) and then over 8 mice. (*B*) Heat maps with the estimated coherence strength for each frequency. Purkinje cell stimulation induced a delayed increase in the lower frequency range, mainly theta band. Sensory stimulation predominantly caused a rapid increase in the lower gamma-band range. This increased coherence in the lower gamma band range could be suppressed by simultaneous optogenetic Purkinje cell stimulation. This suppression was largely absent when the optogenetic stimuli were delayed by 20 ms, indicating the importance of fast cerebellar processing. (*C*) Heat maps showing Granger causality from wS1 to wM1. (*D*) Granger causality for wM1 to wS1. (*E*) Estimated coherence after whisker stimulation (*Left*) and for optogenetic Purkinje cell stimulation alone (*Right*). (*F*) Simultaneous Purkinje cell stimulation enhanced and suppressed the estimated theta- and gamma-band coherence, respectively. These modulations were largely reduced by a 20-ms delay in the onset of Purkinje cell stimulation. The increased theta-band activity may partly reflect a direct effect of Purkinje cell stimulation. Shaded areas indicate SEM (*n* = 8 mice; *P < 0.05, χ^2^ > 3.84, DoC test; *Materials and Methods*).

As we found cerebellar activity to be able to modulate sensory responses in both wS1 and wM1, and that these modulations differentially affected specific frequency ranges, we surmised that cerebellar activity could also affect mutual phase consistency, i.e., coherence, in sensory-related activities between these areas. We reasoned that, although the sensory responses were transient, cerebellar impact on sensory-induced phase consistency could still be relevant given that most of the sensory input reaches wS1 and wM1 during this transient period. As there are particularly strong connections between the deep layers of wS1 and superficial layers of wM1 (11, 12, 25) (*SI Appendix*, Fig. S1*A*), we initially focused on temporal patterns between these layers. Compared to the wS1–wM1 coherence estimated during intertrial intervals, air-puff stimulation of the whiskers triggered a fast increase particularly in the beta- and gamma-band ranges, and to a lesser extent also in the theta range (*SI Appendix*, Figs. S10 and S11). When we combined the sensory stimulation with simultaneous optogenetic Purkinje cell stimulation, we observed a further enhancement of the estimated sensory-induced coherence at the theta band, but also a prominent reduction at the gamma band (Fig. 2*B*, magenta and white arrows, respectively; see also *SI Appendix*, Fig. S12 *A* and *C*). Both of these modifications could be alleviated by delaying the optogenetic stimulation 20 ms relative to the onset of whisker stimulation (Fig. 2 *A*–*E* and *SI Appendix*, Fig. S12 *B* and *D*). The fact that a 20-ms lag between sensory and Purkinje cell stimulation was sufficient to, at least in part, rescue the original amplitude of sensory-evoked signals corroborates the notion that, under normal physiological circumstances, cerebellar modulation of cerebral coherence is probably mediated by a fast pathway. Granger causality can quantify how much of the wM1 signal in a certain frequency band can be causally predicted by the activity of wS1 and vice versa (58). Applying this analysis to the sensory-induced conditions suggests that the deep layers of wS1 and superficial layers of wM1 are both involved in generating the estimated coherence (Fig. 2 *C* and *D*).

A decrease in the common input to wS1 and wM1, as was indeed observed by adding Purkinje cell stimulation to sensory stimulation (Fig. 1), could potentially obfuscate the reliable estimation of the coherence (22). To establish whether the reduction in sensory-induced gamma-band coherence was the sole consequence of a decrease in response amplitude, we also estimated the sensory-induced coherence between the other cortical layers. Most notably, Purkinje cell stimulation did not affect the amplitude of the event-related potential in the superficial layers of wS1 and wM1 (Fig. 1 *H* and *N*). Despite this differential impact on the signal amplitude, the impact of Purkinje cell stimulation on the estimated sensory-induced gamma-band coherence was rather similar when comparing deep wS1 and superficial wM1 versus superficial wS1 and superficial wM1 (*SI Appendix*, Fig. S13 *A* and *B*). From this comparison, we conclude that the estimated changes in transient sensory-induced coherence cannot be solely explained by the reduction of common input to wS1 and wM1 as a consequence of Purkinje cell stimulation.

Estimating the transient coherence between other cortical layers, which are probably less directly coupled (*SI Appendix*, Fig. S1*A*), we observed that the cerebellar impact broadened, now also comprising beta and higher gamma bands (*SI Appendix*, Fig. S13). Here, Granger causality analysis supported the notion that the sensory-induced gamma-band coherence largely starts in the superficial layers of wS1 and, from there, spreads to the deep layers of wS1 (*SI Appendix*, Fig. S14 *A* and *B*) and the deep layers of wM1 (*SI Appendix*, Fig. S14 *C* and *D*). Notably, there was more balance between the deep layers of wS1 and the superficial layers of wM1 (Fig. 2 *C* and *D* and *SI Appendix*, Fig. S14 *C* and *D*), suggesting that sensory-induced coherence is a complex phenomenon involving reciprocal connections between wS1 and wM1. Granger causality analysis of the Purkinje cell-induced theta-band coherence did not systematically reveal a strict directionality, also suggesting a reciprocal involvement of wS1 and wM1.

Thus, experimentally dampening the output of the cerebellar nuclei by enhancing Purkinje cell activity results in changes in the amplitude and temporal characteristics of sensory responses in wS1 and wM1, also affecting sensory-induced coherences between wS1 and wM1. Effects were detected in all cortical layers, with the most specific and reproducible changes estimated to be in the comparison between theta and gamma coherence across wS1 and wM1.

### Cerebellar Impact on Estimated wS1–wM1 Coherence Depends on Behavioral Context.

Next, we wondered whether the estimated wS1–wM1 coherence, as approximation of consistency in the phase relations of sensory response between trials, depended on the acute motor behavior. The interpretation of sensory input from the whiskers is state-dependent, as, for instance, self-motion generates a lot of input that has to be considered when processing whisker sensory input. We therefore sought to study variations in whisker movement between trials and relate these to differences in the estimated coherence and/or its modulation by simultaneous Purkinje cell stimulation. To this end, we made use of air puff-induced reflexive whisker protraction, the amplitude of which is correlated to cerebellar activity (43, 59). Likewise, termination of optogenetic stimulation of Purkinje cells also triggers whisker protraction (39, 43, 59). As a consequence, the combined stimuli induced complex but reproducible whisker movements, during which Purkinje cell stimulation always increased the power of the movement (*SI Appendix*, Figs. S15 *A*–*D* and S16). These findings raise the question as to what extent cerebellar activity controls the interaction between whisker movements and activity in wS1 and/or wM1, and whether the impact of Purkinje cell activity on coherence between wS1 and wM1 depends on the power of the movement.

The estimated coherence between LFP and the power of the whisker movements following air-puff stimulation was altered by simultaneous Purkinje cell stimulation: lower frequencies (delta–theta band) were enhanced, whereas the higher frequencies (beta–gamma band) were suppressed (*SI Appendix*, Fig. S15*E*). The increased LFP–whisker coherence at the delta and theta bands were present throughout wS1 and wM1, whereas the suppression of the beta- and gamma-band coherence was particularly pronounced in the deep layers of wM1 (*SI Appendix*, Fig. S15*F*). In these experiments too, a delay of 20 ms between the onset of whisker and Purkinje cell stimulation mitigated the impact of Purkinje cell stimulation considerably (*SI Appendix*, Fig. S15*F*).

**Fig. 3.**
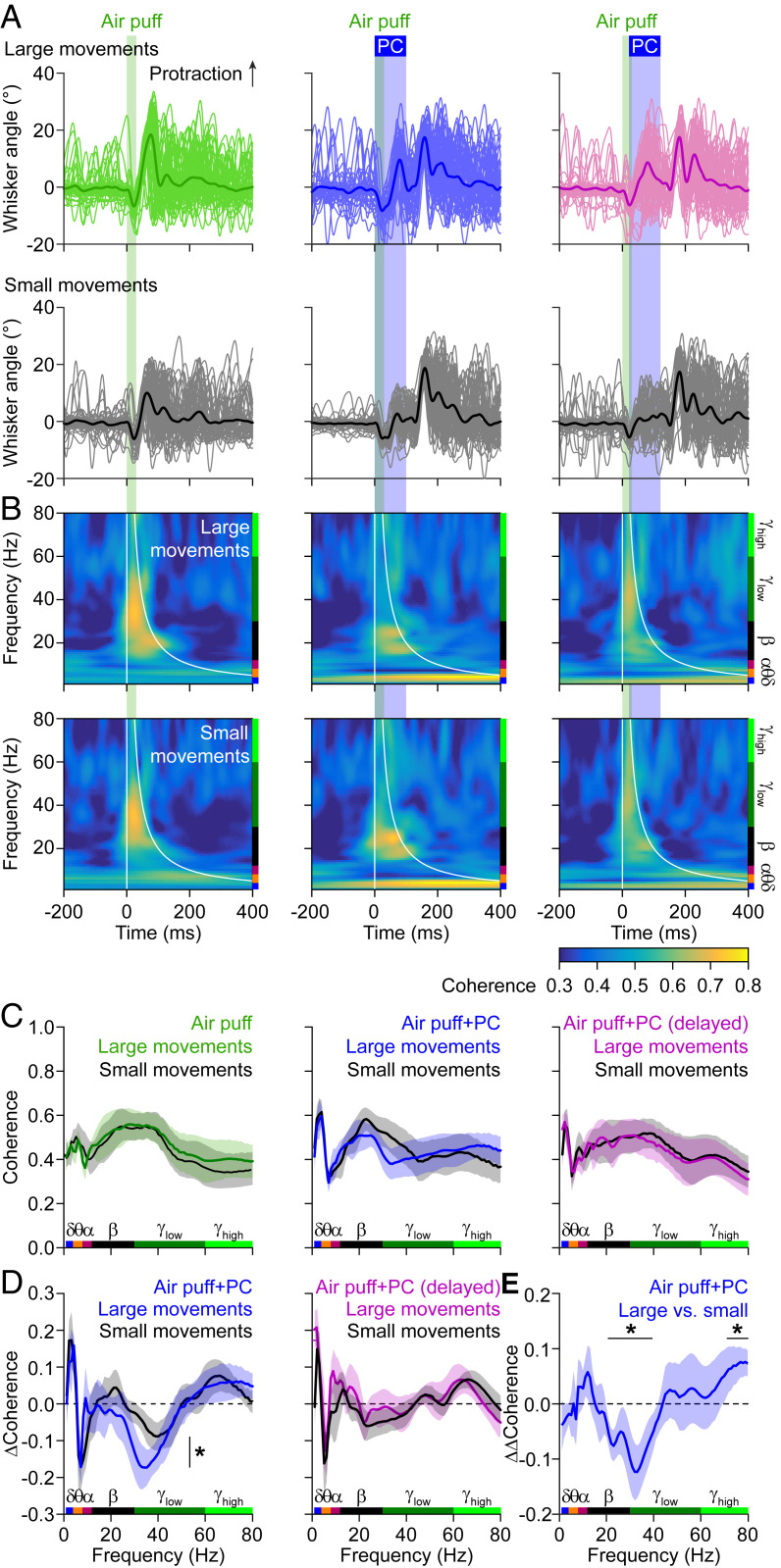
Cerebellar impact on estimated wS1–wM1 coherence depends on behavior. (*A*) Whisker stimulation triggers reflexive protraction. Trials were split between the 50% of the trials with the largest and the 50% with the smallest protractions. (*B*) The estimated coherence between the subgranular layers of wS1 and the supragranular layers of wM1 were only mildly different between the trials with large (*Top*) and small (*Bottom*) movements. (*C*) For each stimulus condition, the averaged coherence spectra are plotted, with colored traces representing the large whisker movements. Note that the difference in beta- and lower gamma-band activity are modulated in opposite fashion when adding optogenetic Purkinje cell stimulation to the air-puff stimulation. (*D*) Accordingly, the impact of optogenetic Purkinje cell stimulation on the estimated sensory-induced wS1–wM1 coherence was stronger during trials with large whisker movements. This difference was statistically significant (DoC test; see *E*). This effect was abolished by a 20-ms delay between the start of whisker and Purkinje cell stimulation. (*E*) The difference in the impact of simultaneous Purkinje cell stimulation (“ΔΔCoherence”) on sensory-induced beta- and gamma-band coherence was significantly larger during the trials with large movements than during those with small movements (DoC analysis). Lines in *C* to *E* indicate averages and the shades SEM (*SI Appendix*, Fig. S17).

To analyze the impact of Purkinje cell stimulation on the correlation between wS1–wM1 coherence and whisker behavior, we made use of the normally distributed intertrial variations in the amplitude of reflexive whisker protractions. First, for each experiment, we singled out the 50% of the trials with the largest reflexive whisker protractions, comparing these to the other 50% (Fig. 3*A*). During air-puff stimulation, the estimated coherence values were lower for the larger movements around the theta band and similar for both categories of movement in the gamma band, while, during combined whisker and Purkinje cell stimulation, the suppressive impact of Purkinje cell stimulation was larger in the beta and gamma bands (Fig. 3 *B*–*E*). Again, this impact was absent when introducing a 20-ms delay between whisker and Purkinje cell stimulation (Fig. 3*D*). We subsequently refined this analysis by subdividing the trials in eight overlapping groups based upon the whisker protraction amplitudes. The coherence changed monotonically across the groups, confirming our initial analysis based on the division in two groups (*SI Appendix*, Figs. S17 and S18). Finally, we were also able to predict the whisker amplitude based on the estimated coherence level using linear regression. When we examined the trials with only sensory stimulation to estimate the protraction amplitude, the optimal coherence predictor was characterized by a negative peak in the lower frequency range (theta and alpha band) as well as a positive peak in the beta range (*SI Appendix*, Fig. S17*E*). During simultaneous whisker and Purkinje cell stimulation, the predictor was more complex, having negative peaks in the theta, beta, and lower gamma bands and a positive peak in the alpha band, while the 20-ms delay condition resulted in a predictor that was more similar to the air puff-only scenario (*SI Appendix*, Fig. S17*E*).

Next, we compared the linear regression analyses between the different conditions and found that, during synchronous whisker and Purkinje cell stimulation, the predictor had the lowest value (*R*^2^ = 0.70 ± 0.12 of the variance in the protraction amplitudes), whereas the predictor had the highest value during trials with only whisker stimulation (*R*^2^ = 0.93 ± 0.02). The trials with delayed Purkinje cell activation had an intermediate value (*R*^2^ = 0.86 ± 0.03; *SI Appendix*, Fig. S17*F*, black bars). Since the predictors of the trials with only whisker stimulation and those with whisker and delayed Purkinje cell stimulation had similar shapes (*SI Appendix*, Fig. S17*E*), we can expect that one of them can partially predict the whisker amplitudes in the other case. To verify this, we applied the whisker-only predictor to the trials with delayed Purkinje cell activation and obtained an impaired, yet still significant, accuracy in predicting the whisker amplitude (*R*^2^ = 0.34 ± 0.14). In contrast, with the same predictor, we could not get any valid prediction in the synchronous activation case (*SI Appendix*, Fig. S17*F*, red bars).

In summary, coherence in cortical activity, an estimate of consistency in phase relations across trials, relates to the behavioral context, i.e., the level of whisker movements. Optogenetic Purkinje cell activation, which affects the estimated coherence in specific ways, impairs this relationship most prominently when the activation coincides within a narrow time window with respect to the sensory stimulus. The cerebellum therefore seems to provide a context-dependent input, operating in specific frequency bands. In contrast, Purkinje cell stimulation in the absence of whisker stimulation does not prominently affect whisker movement until the end of the stimulus (*SI Appendix*, Figs. S16 and S19) (39).

### Regional Heterogeneity in Cerebello-Cerebral Communication.

**Fig. 4.**
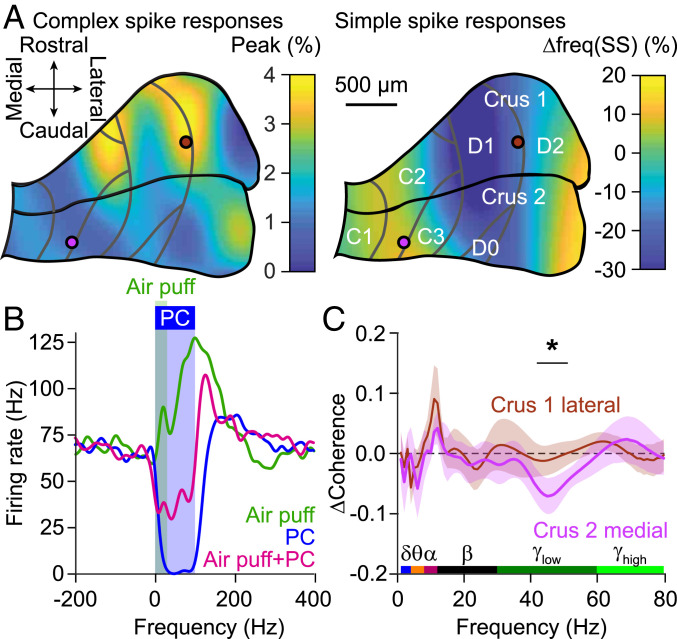
Regional heterogeneity in cerebello-cerebral communication. (*A*) Air-puff whisker-pad stimulation results in bidirectional modulation of Purkinje cell simple spike firing. Heat map illustrates the distribution of the maximal modulation within 80 ms of stimulation, showing a difference between medial and lateral zones. (Modified from ref. 43, which is licensed under CC BY 4.0). Purkinje cells in the C zones project to the interposed nucleus; those in the D zones to the lateral nucleus. Note that whisker stimulation can either increase or decrease the simple spike rate. The gray lines indicate the tentative borders between the cerebellar zones. The two colored circles indicate the approximate positions of the 105-µm-diameter optic fibers. (*B*) Representative recording of a cerebellar nucleus neuron showing increased firing upon air-puff stimulation to the whiskers, a virtually complete block of activity during optogenetic Purkinje cell stimulation, and a strong reduction in firing during combined stimulation. (*C*) Compared to air-puff stimulation in the absence of optogenetic stimulation, stimulation of Purkinje cells in the medial part of crus 2 and those in the lateral part of crus 1 had different effects specifically on sensory-induced gamma-band coherence (*SI Appendix*, Figs. S20–S22).

The direction of Purkinje cell modulation upon whisker stimulation is related to the stimulus location within crus 1 and 2 (43). More specifically, whereas increased simple spike firing is most prominent in medial crus 2, that of the complex spikes, which may facilitate execution of touch-induced whisker protraction, is more robust in lateral crus 1 (43). Given this differential distribution in whisker-related Purkinje cell activity (Fig. 4*A*) and the variations in the anatomical projection from the cerebellum to S1 and M1 (36), we hypothesized that the changes in coherence described above depend on the specific stimulus location. To study the impact of the precise stimulus location on coherence between wS1 and wM1 at a high spatial resolution, we used low-powered optic fibers with a relatively small diameter of 105 µm (delivering 0.2 mW vs. 7.0 mW by the 400-µm-diameter fiber), selectively targeting the medial or lateral parts of crus 1 and 2, which project to the interposed and the lateral cerebellar nucleus, respectively (60). The small fibers delivered sufficient power to trigger robust reductions in neuronal activity in the cerebellar nuclei and trigger whisker movements related to rebound firing in these nuclei (*SI Appendix*, Fig. S20 *A*–*C*), while the illuminated volumes were small enough to minimize cross-talk between medial and lateral stimulus locations (*SI Appendix*, Fig. S20 *D* and *E*). Simultaneous sensory and optogenetic stimulation resulted in similar Purkinje cell responses as sensory stimulation alone (*SI Appendix*, Fig. S20*F*). The impact of Purkinje cell stimulation typically resulted in strong reduction of sensory-evoked firing in the cerebellar nuclei (Fig. 4*B*), with the remaining activity probably resulting from mossy fiber collateral innervation of the cerebellar nuclei (57, 59) (*SI Appendix*, Fig. S20*G*).

Consistent with the reduced power of optogenetic stimulation, stimulation with a smaller fiber at any of the four locations resulted only in a marginal impact on the amplitude of the event-related potential in wS1 and wM1 (*SI Appendix*, Figs. S21 and S22 *A* and *B*). In other words, our experimental manipulation affected only a small fraction of the cerebellar output, not enough to make a significant impact on the level of neural activity in wS1 and wM1. However, we did observe differential impacts of localized Purkinje cell stimulation on the estimate of the sensory-induced wS1–wM1 coherence. Consistent with the hypothesis described above, we found prominent differences in the impact of optogenetic stimulation during whisker stimulation between medial crus 2 and lateral crus 1 (Fig. 4*C* and *SI Appendix*, Figs. S22*C* and S23). More specifically, Purkinje cell stimulation in medial crus 2 showed a stronger impact on the estimated sensory-induced coherence in the gamma band than that in lateral crus 1 (Fig. 4*C*). Instead, we found no significant differences at the lower frequency bands. These data on regional heterogeneity are consistent with the prominent dependency of gamma-, but not theta-, band coherence on behavioral context (Fig. 3), and they are also in line with differential Purkinje cell modulations during different forms of adaptive and reflexive whisking behavior (43).

### Dissecting the Impact of Neural Pathways Using a Laminar Model.

**Fig. 5.**
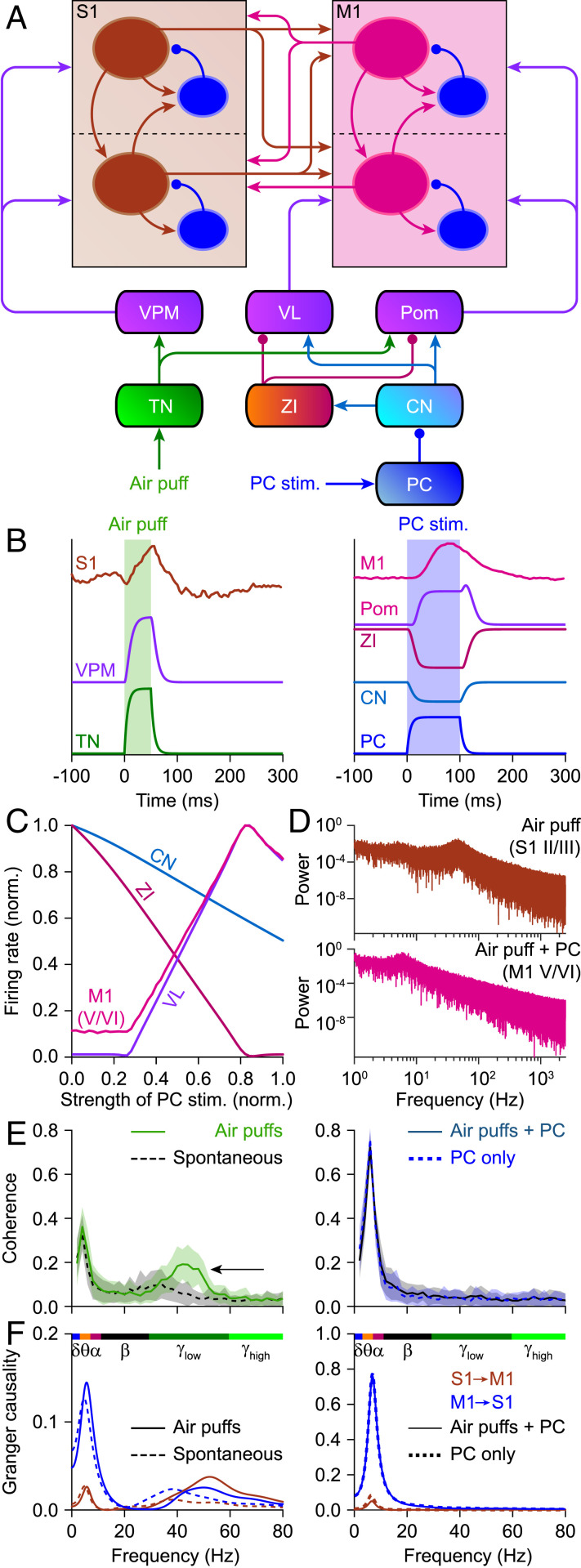
Laminar model. (*A*) Schematic representation of the connections present in the computational model we used to study cortical coherence in silico. CN, cerebellar nuclei; PC, Purkinje cells; TN, sensory trigeminal nuclei; ZI, zona incerta. (*B*) Temporal evolution of the normalized firing rate activity for different populations in the presence of simulated air puff (*Left*) or optogenetic activation of Purkinje cells (*Right*). All populations display short transients in both cases. (*C*) Impact of sustained Purkinje cell stimulation on the stationary firing rates of four different areas. (*D*) Power spectra of the activity of the excitatory populations of supragranular S1 during air-puff stimulation (*Top*) and of subgranular M1 during simultaneous air-puff and Purkinje cell stimulation (*Bottom*), indicating suppression of gamma and enhancement of theta and alpha bands for Purkinje cell stimulation. (*E*) Coherence between S1 and M1 during stimulation of the trigeminal nuclei (simulating air puffs), the stimulation of Purkinje cells, and the combination of both stimuli. Trigeminal stimulation increased coherence in the gamma band, while Purkinje cell stimulation promoted theta-band coherence. Adding Purkinje cell stimulation to trigeminal stimulation cancelled the increased gamma-band coherence. Lines represent means and shaded areas SD. (*F*) Granger causality analysis revealing a largely bidirectional flow between S1 and M1 during gamma-band coherence. Lower frequencies predominantly originated from M1. Note that *C* to *E* were obtained using steady-state input to sensory trigeminal nuclei and/or Purkinje cell (*SI Appendix*, Figs. S24–S26).

We recapitulated the experimental findings by adapting a large-scale computational model of the laminar cortex and subcortical structures (17) to the anatomical pathways relevant for whisker and Purkinje cell stimulation (Fig. 5*A*). We mimicked whisker stimulation by trigeminal activity, which percolated through the network, as did Purkinje cell stimulation (Fig. 5*B*). An impulse-like input induced dampened oscillations, which could be prolonged by introducing noise to the network dynamics (*SI Appendix*, Fig. S24). This demonstrates that even seemingly instantaneous changes, as also evident in the sensory responses in mice, can cause longer-lived temporal features in the signal (see also the fluctuations in *SI Appendix*, Fig. S8*B*). For the following analyses, we used steady-state input in a network in the presence of noise. Increasing the intensity of sustained Purkinje cell stimulation had differential effects on different regions, reflecting the contributions of excitatory and inhibitory connections between them (Fig. 5*C*). The power spectra of the stationary signal revealed increased gamma power on supragranular S1 activity in response to trigeminal activity, while Purkinje cell stimulation led to increased theta power on subgranular M1 (Fig. 5*D*). Stimulating the trigeminal nucleus, simulating whisker input, induced increased coherence in theta and lower gamma range, the latter being inhibited by simultaneous Purkinje cell stimulation (Fig. 5*E*, black arrow), mimicking the experimental data (Fig. 2). Granger causality analysis of the model data revealed that the sensory-induced gamma-band coherence was approximately symmetrical between wS1 and wM1, while the theta-band coherence caused by Purkinje cell activity was largely inflicted upon wS1 by wM1 (Fig. 5*F*). The balance between S1 and M1 in causing sensory-induced gamma-band coherence proved to be particularly dependent on the reciprocal connectivity between the superficial layers of S1 and M1 (*SI Appendix*, Fig. S25). Moreover, our model also confirmed that Purkinje cell stimulation can be responsible for the enhancement of theta coherence between cortical areas. Given the prominent similarity of the modeled and experimental datasets under various conditions, we next looked at the potential relevance of the different thalamic hubs that were not directly tested in the experiments. These modeling data suggest that the ventrolateral nucleus (VL), but also the ventroposterior medial nucleus (VPM) of the thalamus, had a stronger impact than the medial posterior nucleus (Pom) in mediating the impact of cerebellar activation onto cortical coherence between wS1 and wM1 during sensory stimulation (*SI Appendix*, Fig. S26), which is in line with the distribution of afferents from cerebellum and trigeminal nucleus to the thalamus (3).

## Discussion

Tactile exploration of the world requires acute motor control with fast integration of sensory feedback. This is how one adapts the grasp force to keep hold of a slipping cup, or how a tennis player adapts his stroke to side wind. To study fast sensorimotor integration in mammals, exploration by whisker touch has become a popular model system (1–3). In line with the behavioral relevance to mice, neural control of the facial whiskers is complex, involving synergistic control of cerebellum and neocortex (35). Here, we found that transient disruptions of cerebellar output, induced by optogenetic stimulation of Purkinje cells, could disinhibit sensory LFP responses to whisker stimulation in wS1 and wM1 and differentially modulate sensory-induced wS1–wM1 theta- and gamma-band activity, as estimated using coherence analysis. The impact of Purkinje cell stimulation on the coherence in the gamma, but not theta, range depended on the acute behavior as well as the precise location in the cerebellar cortex (*SI Appendix*, Fig. S27).

Purkinje cell stimulation, when performed simultaneously with whisker stimulation, could reduce the amplitude of the LFP signal in the deep layers of wS1 and wM1 (Fig. 1), and this was paired with a reduction in the gamma-band coherence between wS1 and wM1 (Fig. 2). These two phenomena could be related, as it has been demonstrated that a reduction in the amplitude of the neural signal can lead, via a reduced signal-to-noise ratio, to an apparent drop in coherence level (22, 61). Although we cannot exclude that this effect contributed to the reduced sensory-related gamma-band coherence observed upon simultaneous Purkinje cell stimulation, this is unlikely to be the prime explanation for our observations. Comparing the coherence between different layers of wS1 and wM1 (*SI Appendix*, Fig. S13) and between the large (Fig. 2) and the small optic fiber (Fig. 4 and *SI Appendix*, Fig. S22) demonstrates that there is no apparent coupling between the impact of optogenetic Purkinje cell stimulation on the amplitude of the signal in wS1 and wM1 and the impact on the estimated wS1–wM1 coherence. This is also in line with reduced gamma-band coherence between the power of whisker movement and wS1 and wM1, respectively, despite the stronger power of whisker movement as a consequence of Purkinje cell stimulation (*SI Appendix*, Fig. S15). We therefore conclude that the signal-to-noise ratio of our intracortical LFP recordings (*SI Appendix*, Fig. S3) is of sufficient value to enable estimating the coherence, even at reduced signal amplitudes.

The sensory-induced LFP responses in wS1 and wM1 are short-lived (Fig. 1 *E* and *K* and *SI Appendix*, Figs. S2–S5). Current source density, phase-difference, and Granger analyses revealed, in line with the known anatomical connections (3, 40), that, after whisker stimulation, the earliest response appeared in the granular layer of wS1, after which it spread to the other layers of wS1 as well as to wM1 (*SI Appendix*, Figs. S5, S9, and S14). The phase of the ongoing signal can be of relevance for the impact of synaptic input (21), and, as it can be expected that most of the sensory input arrives during the 200 ms during which the sensory-induced LFP signal lasts (Fig. 1), the transient nature of the sensory-induced responses is not contradicting the impact of the phase of the response, and thus warrants analysis of coherence or phase consistency, also under our experimental conditions. This notion is in line with the concept that the cerebellum is often considered as crucial when it comes to the accurate timing of movements (41, 62, 63). In line with this, cerebellum-induced modulations of the LFP signal in wS1 and wM1 were only partially related to the rate of spiking (*SI Appendix*, Fig. S2). In particular, the positive LFP peak could not be readily matched with multiunit activity, suggesting that it affected subthreshold currents and spike timing rather than spike frequency.

The impact of Purkinje cell stimulation on individual LFP responses in wS1 and wM1 can be readily explained by the anatomical pathways involved. Whisker sensory information is rapidly relayed to wS1 via lemniscal and extralemniscal pathways passing by the thalamic VPM nucleus (3, 12, 40, 64–67). Optogenetic reduction of cerebellar output did not affect the initial excitation in wS1, but it did alter subsequent current spread (*SI Appendix*, Fig. S5 *A*–*C*). Next to the direct excitatory output from the cerebellar nuclei to the thalamus, there is also a prominent inhibitory projection via the zona incerta (3, 65). Indeed, inhibitory activity of the zona incerta has been implicated in subcortical suppression of whisker sensory input during self-motion (3, 64, 66, 67), which highlights its role as important intermediate between cerebellum and neocortex. The zona incerta sends GABAergic projections in particular to Pom, which also receives direct inputs from the trigeminal and cerebellar nuclei, next to its inputs from wS1 and VL (3, 67, 68). Thus, during whisker motion, the cerebellum could, together with wM1 (66), activate the zona incerta that in turn suppresses thalamic activity. Recently, we described that the impact of cerebellar output depends on the behavioral state of the animal (43, 69). Thus, we hypothesize that differential cerebellar output, depending on the behavioral state, can modulate sensory responses in wS1 and wM1, thereby adjusting sensory feedback to acute motor activity of the whiskers and/or the head.

In wS1, Pom terminals can be found mainly in layers I and V (70), in line with our finding of an impact of cerebellar stimulation on the subgranular layers. In wM1, Purkinje cell stimulation resulted in inhibition of the sensory response, which again is compatible with the projections from Pom. We found the impact of Purkinje cell activation to be region-specific, in line with the unequal strengths of projections to S1 and M1: the ratio between projection strengths to S1 and M1 is in favor of M1 in the C1 zone of the cerebellar cortex, whereas it is biased toward S1 in the C3 and D2 zones (36). In addition, there are also variations in sensory representation across crus 1 and crus 2 (43, 53, 55, 71). Moreover, in all cases, a brief delay between sensory and Purkinje cell stimulation reduced the cerebellar impact substantially, which follows our experimental finding that a fast input from the cerebellum appears to be essential for the normal responses in wS1 and wM1.

In sensorimotor cortex, coherent LFP oscillations have been observed in various vertebrates, including rodents and primates. In primates, gamma band-synchronized oscillations in the arm areas of motor and somatosensory cortex have been linked to arm movements, although they also occurred spontaneously (46, 72, 73). Also in rodents, correlations between sensorimotor areas relate to concrete motor functions and planning. Areas wS1 and wM1 in mice display context-dependent coherent activity, reflecting motor feedback to sensory processing (74). As in primates, gamma-band coherence in rodents is linked mostly to movement generation, such as during active whisking as in the current study, but it can also be generated spontaneously (75, 76). In our study (*SI Appendix*, Fig. S11), the coherence during spontaneous activity occurred predominantly in the lower frequency bands.

Correlated activity between S1 and frontal areas such as M1 and the prelimbic cortex has also been found to play a role in functions other than movement, such as processing of working memory, with strong theta coherence in the case of S1 and prelimbic cortex (77, 78). Different functions of cortical coherence have also been described in primates. For instance, the primate primary sensory and motor cortices display prominent coherence in the beta band linked to movement suppression (79), while other rhythms are involved in sensorimotor integration, displaying specific spatiotemporal patterns (26). Communication between sensory and motor cortex constitutes a bidirectional channel, with motor output signals from motor areas being received by S1 before sensory feedback arrives to these areas, therefore potentially enabling anticipatory activity (80). Our results extend these findings by characterizing the impact of cerebellar structures on transient S1–M1 coherence, in particular in the theta and gamma bands.

The impact of Purkinje cell stimulation on estimates of coherence between wS1 and wM1 showed that cerebellar output can bidirectionally modulate sensory-induced coherence in the theta and gamma bands. However, the finding that only the impact in the gamma range depended on ongoing behavior and on the precise location of stimulation in the cerebellar cortex raises the possibility that the cerebellum exerts its functional effects in the cortex mainly through a more high-frequency mode of operation, in line with its high intrinsic firing rate (81). Moreover, these findings also suggest that the cerebellum may better control motor behavior by temporarily downgrading cortical activity levels with sensory-relevant signals rather than enhancing them. These implications agree with the high-frequency mode of simple spike activity and modulation that take place during the preparation and execution of motor coordination (43, 59, 82, 83). Indeed, we found that the suppressive impact of Purkinje cell stimulation on gamma-band activity was greater during larger movements, and this impact could be specifically linked to the Purkinje cells in medial crus 2. Finally, our data also align well with the differential frequencies of coherences that are implicated during the different stages of motor planning and execution (26, 34).

The impact of cerebellar activation on individual LFP signals in wS1 or wM1 as well as that on the coherence between these signals could be replicated well by our modeling work. We built our computational model on cerebellar modulation of cortical interactions by expanding our existing model on cortico-cortical and thalamo-cortical interactions (17, 32). The model, the connectivity of which is constrained by realistic anatomical routes, suggests that Purkinje cell activity triggers a disinhibitory effect via the zona incerta, which in turn mediates both the suppression of gamma coherence and the enhancement of theta coherence between S1 and M1. In agreement with the experimental data, the model revealed that sensory-induced gamma-band coherence involved mainly signals originating from S1, but with a substantial contribution of M1. The impact of M1 on gamma-band coherence depended on the extent of reciprocal connectivity of S1 and M1, rather than on thalamic activity (*SI Appendix*, Fig. S25). Changing the connectivity at the level of the thalamus could alter the coherence at lower frequencies, with the stronger connectivity being more in line with the experimental data, but had little impact on the directionality of coherence (*SI Appendix*, Fig. S26). Given the similarities between the modeling and experimental outcomes, even explaining counterintuitive findings, the current model may well provide detailed and valid predictions as to how the cerebellum may influence the different layers and areas of the cerebral cortex under a wider and richer variety of physiological behaviors.

## Materials and Methods

### Animals.

Experiments were performed on heterozygous transgenic mice expressing the light-sensitive cation channel channelrhodopsin-2 under the Purkinje cell-specific *Pcp2* promoter [Tg(Pcp2-cre)2MPin;Gt(ROSA)26Sor^tm27.1(CAG-COP4*H134R/tdTomato)Hze^] on a C57BL6/J background (57). We used 14 males and 12 females aged between 10 and 34 wk. The mice were kept in a vivarium with controlled temperature and humidity and a 12/12 h light/dark cycle. The animals were group-housed until surgery and single-housed afterward. Ethical approval was granted prior to the start of the experiments from the national authority (Centrale Commissie Dierproeven, The Hague, The Netherlands; license no. AVD101002015273) as required by Dutch law, and all experiments were performed according to institutional, national, and European Union guidelines and legislation.

### Surgery, Stimulation, and Recording.

The mice received a pedestal to allow head fixation in the recording setup and underwent one to three craniotomies. After 3 d of recovery, the mice were habituated to the setup on at least three consecutive days, with increasing habituation times (10 to 120 min). Recordings were performed in awake mice (*SI Appendix*, *Supplementary Methods*).

### Coherence Analysis.

Phase-coherence analysis was computed using the FieldTrip toolbox (*SI Appendix*, *Supplementary Methods*). LFP snippets of 5 s pre- and 5 s poststimulus were used to calculate the coherence spectrum per trial. If necessary, 50 Hz line noise was removed by power spectrum normalization (84). Next, the coherence in a frequency-dependent time window (two oscillations for a given frequency) after stimulus onset was averaged per frequency. The effect of Purkinje cell activation on sensory-triggered coherence was estimated by subtracting the averaged air puff-induced coherence from the coherence evoked by air puff with photostimulation [difference of coherence (DoC) analysis; *SI Appendix*, Fig. S10]. Granger causality analysis was carried out using the FieldTrip toolbox with the same preprocessing.

### Computational Model.

The computational model used is based on a previous work (17), with minimal variations in the cortical parameters based on observed anatomical and physiological properties of wS1 and wM1 in mice and the addition of trigeminal nucleus, thalamic nuclei, and cerebellum (*SI Appendix*, *Supplementary Methods*).

### Experimental Design and Statistical Analysis.

We considered *P* ≤ 0.05 as significant unless Benjamini–Hochberg correction for multiple comparisons was applied (*SI Appendix*, Table S1). Two-tailed testing was used for all statistical analyses. *N* indicates the number of mice; *n* indicates the number of stimuli/recordings (*SI Appendix*, *Supplementary Methods*).

## Supplementary Material

Supplementary File

## Data Availability

Electrophysiological recording data and whisker movement traces have been deposited in Zenodo (DOI: 10.5281/zenodo.4312336).
