# Supplementary material for: Cerebellar Purkinje cells can differentially modulate coherence between sensory and motor cortex depending on region and behavior
Source: Proc Natl Acad Sci U S A. 2020 Dec 22;118(2):e2015292118. doi: 10.1073/pnas.2015292118 (PMC7812746; doi:10.1073/pnas.2015292118)
Supplement: Supplementary File [file pnas.2015292118.sapp.pdf]

PNAS
www.pnas.org

Supplementary Information for

Coherence bands between primary sensory and motor cortex can be differentially modulated by cerebellar Purkinje cells in a region-specific and behavior-dependent manner

Sander Lindeman, Sungho Hong, Lieke Kros, Jorge F. Mejias, Vincenzo Romano, Robert Oostenveld, Mario Negrello, Laurens W.J. Bosman and Chris I. De Zeeuw

Laurens Bosman
Email: l.bosman@erasmusmc.nl
Mario Negrello
Email: m.negrello@erasmusmc.nl

This PDF file includes:

Supplementary Methods
Figures S1 to S27
Table S1
SI References

Supplementary methods

Surgery. All surgical procedures were performed under anesthesia (2-5% isoflurane in 1 l/min oxygen) in combination with treatment of surgical pain, giving 5 mg/kg carprofen (“Rimadyl”, Pfizer, New York, NY, USA), 1 µg bupivacaine (Actavis, Parsippany-Troy Hills, NJ, USA) and 50 µg/kg buprenorphine ("Temgesic”, Indivior, Richmond, VA, USA). In addition, the mice received 1 µg lidocaine (Braun, Meisingen, Germany) subcutaneously at the surgical sites prior to the start of the surgery. During anesthesia, the body temperature was maintained at 37 °C by a feed-back controlled heating pad.

For the placement of a pedestal, a part of the skin was removed and the skull was cleaned and treated with phosphoric acid to ensure all membranes were removed. Next, the exposed skull was treated with Optibond adhesive (Kerr Dental, Orange, CA, USA) and the mice received a magnetic pedestal that was placed on the skull between the eyes and secured with Charisma (Kerr Dental). Next, up to three craniotomies were performed allowing access to the whisker part of the left primary somatosensory (wS1, relative to bregma: 3.5 mm mediolateral and -1.5 mm anteroposterior) and motor cortex (wM1, relative to bregma: 1.5 mm mediolateral and 1.0 mm anteroposterior) and the right cerebellar hemisphere, each surrounded by a recording chamber made out of Charisma. The exposed dura was covered with tetracycline-containing ointment (Terra Cortril; Pfizer, New York, NY, USA) and the recording chambers were sealed with a silicon polymer (Kwik-Cast, WPI, Sarasota, FL, USA) and covered with bone wax (Ethicon, Somerville, NJ, USA). The animals were given three days of recovery after the surgery before they were habituated to the setup on at least three consecutive days with increasing habituation times (from approx. 10 min the first session to approx. 2 h the last session).

Electrophysiology and stimulation. All recordings were made in awake, head restrained mice. LFP recordings were made in wS1 and wM1 using 16 channel, single shaft silicon probes with an inter-electrode distance of 100 µm (R = 1.5-2.5 MΩ, A1x16-5mm-100-177-A16, NeuroNexus Technologies, Ann Arbor, MI, USA). Each silicon probe was equipped with its own reference, placed in close proximity to the recording site. The two probes shared the same ground, which was placed either in the agar covering the recording sites or in the agar covering the cerebellar craniotomy. Single unit activity of putative cerebellar nuclei neurons was measured using extracellular quartz-coated platinum-tungsten fiber electrodes (R = 2-5 MΩ;

80 µm outer diameter; Thomas Recording, Giessen, Germany) placed in a rectangular matrix (Thomas Recording) with an inter-electrode distance of 305 µm. All electrodes were connected to a PZ5 NeuroDigitizer (Tucker-Davis Technologies). The signals were amplified, 1-6,000 Hz filtered, digitized at 24 kHz and stored using a RZ2 multi-channel workstation (Tucker-Davis Technologies).

Spike times from single-unit recordings were retrieved off-line using Spiketrain (Neurasmus BV, Rotterdam, The Netherlands). Recorded neurons were classified as putative cerebellar nuclei neurons if they were recorded at a depth of at least 1700 µm from the cerebellar surface and if the recording contained only a single type of action potentials typically showing both negative and positive parts, what differentiated them from Purkinje cell simple spikes. Recording locations were established by a combination of the entry points of the electrodes and post-mortem histology. Cerebellar nucleus neurons were recorded in the interposed and the lateral nucleus (Fig. S1D).

Optogenetic stimulation of the cerebellum occurred contralateral to the neocortical LFP recording sites using 470 nm LED drivers (M4703F, ThorLabs. Newton, NJ, USA) connected to a 4-channel LED driver (DC4104, ThorLabs) and optic fibers with diameters of 400 (Figs. 1-3) or 105 µm (Fig. 4) (ThorLabs). The 400 µm fibers were placed just above the dura of the cerebellum. The 105 µm fibers were adapted for insertion into the rectangular electrode matrix by removing the cladding for ~15 cm and grinding the tip under microscope guidance. In each experiment, we either used large of small fibers. Of both groups, we recorded 8 mice to study the interaction between sensory and Purkinje cell stimulation. Of the group of mice recorded during stimulation with the small fibers, one mouse was excluded from parts of the analysis due to excessive noise during a substantial section of the recording. Unless stated otherwise, photostimulation was applied as 100 ms pulses with a power of 7.0 mW (400 µm fiber) or 0.2 mW (105 µm fiber). Sensory stimulations consisted of 30 ms air puffs at 1 bar directed at the mystacial macrovibrissae ipsilateral to cerebellar and contralateral to neocortical recording sites, using a MPPI-2 pressure injector (Applied Scientific Instrumentation, Eugene, OR, USA). The nozzle was positioned to minimize stimulation of the eye or ear. Stimuli were presented at 0.25 Hz in pseudorandom order.

Analysis of LFP signal. Before any analysis was done on the LFP data, the raw traces were normalized using the z-score function in MATLAB (MathWorks, Nattick, MA, USA). The current source density analysis was performed in custom written MATLAB routines using the Kernel Source Density Method as

described in (1); see https://github.molgen.mpg.de/MPIBR-coattia/MatlabMain/tree/master/behaviorAnalysis/code/functions/kCSDv1. To extract multi-unit activity from the silicon probe recordings, a 1 kHz high-pass filter was used to filter out any LFP components. Then, events were detected either directly in the high-pass filtered signal or in its derivative, using a manually set threshold.

Spectrographs (Fig. S8) were computed from data downsampled to 1,000 Hz, with FFT length of 256 samples. The data was pre-filtered to reduce zero frequency component. We employed MATLAB's filter designer and filtered the data as follows:

```
LenFFT = 256; fs = 1000;
hDCB = fdesign.highpass('fst,fp,ast,ap', fs/(2*LenFFT), fs/LenFFT, 50, 0.05, fs);
HdSOSdcb = design(hDCB, 'ellip', 'MatchExactly', 'both');
q = filtfilt(HdSOSdcb.sosMatrix,HdSOSdcb.ScaleValues,q);
```

Coherence analysis. The phase coherence analysis was computed using the FieldTrip toolbox (1). For this, LFP snippets of 5 second pre- and 5 second post-stimulus were used to calculate the coherence spectrum per trial. If necessary, line noise at 50 Hz was removed first from the waveforms by fitting a PSD around the time of the peaks of the power spectrum and then filtering the signal with the inversed square root of this function (2). Next, the coherence in a frequency-dependent window (2 * 1/frequency) after stimulus onset was averaged per frequency to perform the further analysis on. The effect of optogenetic Purkinje cell activation on the sensory triggered wS1-wM1 coherence was determined by subtracting the averaged air puff induced coherence from the air puff with optogenetically evoked coherence. To test for differences between the conditions, the difference of coherence test was used, as described by Amjad et al. (3). In short, the Fisher transform ($\tanh^{-1}$) was applied on the coherence and this was compared to a χ^2-distribution with k - 1 degrees of freedom, where k is the number of conditions that were tested (in all cases k = 2). The 95% confidence limit was then determined using $\chi^2_{(0.05;1)} = 3.84$. The significant frequencies are indicated in the difference of coherence figures using lines and asterisks.

Here we provide a brief explanation for the coherence and its information theoretic implication, mostly following Borst and Theunissen (4). First, we consider two time series *x*(*t*) and *y*(*t*) and denote their Fourier transformation by $X(\omega)$ and $Y(\omega)$. The coherence between them is given by

$$C_{XY}(\omega) = \frac{\left|\widehat{C}_{XY}(\omega)\right|^2}{\left|\widehat{C}_{XX}(\omega)\right|^2 \left|\widehat{C}_{YY}(\omega)\right|^2},$$

where $\widehat{C}_{XY}(\omega) = \langle Y^*(\omega) X(\omega) \rangle$.

Now we will explain the coherence is approximately the information theoretic measure of entanglement via a Gaussian channel. In this case, *x*(*t*) and *y*(*t*) are related by a linear relationship as

$$y(t) = h(t) \star x(t) + \zeta(t)$$

where $\star$ denotes convolution, *h*(*t*) a transfer function, $\zeta(t)$ the Wiener process following the Gaussian statistics, uncorrelated to *x*(*t*). Then, the mutual information between *x*(*t*) and *y*(*t*) is

$$I(X;Y) = \int d\omega \log(1 + R(\omega)).$$

where $R(\omega)$ is a signal-to-noise ratio for the optimal estimation of $Y_{est}(\omega)$ of $Y(\omega)$ from $X(\omega)$. From the linear relation, it turns out

$$Y_{est}(\omega) = H(\omega) X(\omega),$$

$$H(\omega) = \frac{\langle X^*(\omega) Y(\omega) \rangle}{\langle X^*(\omega) X(\omega) \rangle} = \widehat{C}_{YX}(\omega) / \widehat{C}_{XX}(\omega).$$

Then, the signal-to-noise ratio is

$$R(\omega) = \frac{\langle Y_{est}(\omega) Y^*_{est}(\omega) \rangle}{\langle N(\omega) N^*(\omega) \rangle} = \frac{C_{XY}(\omega)}{1 - C_{XY}(\omega)},$$

where $N = Y - Y_{est}$, and the mutual information becomes

$$I(X;Y) = -\int d\omega \log(1 - C_{XY}(\omega)).$$

Therefore, the coherence $C_{XY}(\omega) \approx -\log(1 - C_{XY}(\omega))$ is approximately the mutual information conveyed through each frequency band of this channel, particularly when the coherence is small.

Granger causality. Here we provide a similar information theoretic explanation for the Granger causality, mostly based on ref. (5). Here we consider the discretized version of *x*(*t*) and *y*(*t*) as

$$\begin{aligned} x_{t+1} &= \sum_{s=0} a_{x,s}\, x_{t-s} + \zeta_{t+1}, \quad \mathrm{var}(\zeta) = \Sigma_x, \\ x_{t+1} &= \sum_{s=0} a_{xx,s}\, x_{t-s} + a_{xy,s} y_{t-s} + \zeta_{x,t+1}, \\ y_{t+1} &= \sum_{s=0} a_{yx,s}\, x_{t-s} + a_{yy,s} y_{t-s} + \zeta_{y,t+1}, \\ \Sigma_{zw} &= \mathrm{cov}(\zeta_z, \zeta_w), \quad z, w\colon x \text{ or } y. \end{aligned}$$

The transfer entropy from *y(t)* to *x(t)*

$$T_{y\to x} = I(x_{t+1}; y_t | x_t) = H(x_{t+1}|x_t) - H(x_{t+1}|x_t, y_t)$$

measures the directional information flow (6). Again, in the case of the Gaussian channels, it turns out

$$T_{y\to x} = \frac{1}{2} F_{y\to x}, \quad F_{y\to x} = \log\left(\frac{\Sigma_x}{\Sigma_{xx}}\right),$$

where $F_{y\to x}$ is the total Granger causality.

Again, we write the Granger causality as a sum of the contribution from each frequency band,

$$F_{y\to x} = \int d\omega\, f_{Y\to X}(\omega),$$

where $f_{Y\to X}(\omega)$ can be obtained again via Fourier transformation of the linear relation. For example,

$$\begin{pmatrix} X(\omega) \\ Y(\omega) \end{pmatrix} = \boldsymbol{K}(\omega) \begin{pmatrix} Z_X(\omega) \\ Z_Y(\omega) \end{pmatrix}, \qquad \boldsymbol{K}(\omega) = \begin{pmatrix} A_{XX} & A_{XY} \\ A_{YX} & A_{YY} \end{pmatrix}^{-1},$$

where $A_{\bullet\bullet}$ and $Z_\bullet$ are the Fourier transformation of $a_{\bullet\bullet}$ and $\zeta_\bullet$, respectively. From this, we get (5, 7)

$$f_{Y\to X}(\omega) = \log \frac{\widehat{C}_{XX}}{\Sigma_{XX}|K_{XX}|^2}$$

which is also (a half of) the transfer entropy transmitted through each frequency band of the channel.

Whisking behavior. Whisker movements were recorded with a high-speed video camera (frame rate 1,000 Hz; A504k camera, Basler, Ahrensburg, Germany), using a custom-made LED panel (λ = 640 nm) as back-light. All whiskers were kept intact. Whisker movements were tracked off-line using the BIOTACT Whisker Tracking Tool (BWTT) with the sdGeneric, stShapeSpaceKalman, ppBigExtractionAndFiltering, and wdIgorMeanAngle plugins (http://bwtt.sourceforge.net) (8). Briefly, we first determined the position of the snout in each frame semi-automatically by fitting a template to the snout. After masking the snout and subtracting the unmoved background from each frame, the whiskers were traced in a radial approach. The algorithm detected edges in the frame in consecutive concentric snout-shaped masks around the actual snout mask. Ultimately, we detected the start and end nodes of the fitted line segments, and calculated the

angles of the whiskers from these values. The final BWTT result provided us with the angles of all detected whiskers per video frame. To relate the angles across frames to the tracks, we wrote an algorithm that predicts track values in consecutive frames based on the position and velocity in the angular value as well as the y-position of the last video frames (9, 10). The predicted track values for the next frame were compared with the detected values in the next frame and were assigned according to a minimum deviation approach between them. Finally, the mean angle per frame was calculated from the individual whisker traces.

Regression analysis for coherence and whisker protraction. We first identified the range of the mean-subtracted protraction amplitudes $[A_1, A_{max}]$, and formed eight subranges $[A_1, A_3]$, $[A_2, A_4]$, ..., $[A_8, A_{10} = A_{max}]$ where $A_n = A_1 + (n-1)(A_{max} - A_1)/9$. We divided the trials in eight groups based on which subrange the protraction amplitudes are in. For each group, we computed the coherence for frequencies, *v* = 1, 2, ..., 80 Hz. By summing the coherence with the frequency-dependent time bin, we got the protraction amplitude-dependent coherence matrix C_{iv} where *i* = 1...8. With the average protraction amplitude of each group, r_i (*i* = 1...8), we solved the least square problem,

$$\hat{\boldsymbol{v}} = \min_v E(\boldsymbol{v}), E(\boldsymbol{v}) = \|\boldsymbol{r} - \boldsymbol{C}\boldsymbol{v}\|^2$$

for a predictor vector $\hat{\boldsymbol{v}}$.

To prevent overfitting, we used the principal component regression. Briefly, we first found the singular value decomposition of $\boldsymbol{C} = \boldsymbol{U}\boldsymbol{S}\boldsymbol{V}^{\dagger}$ and found the reduced-rank matrix $\widehat{\boldsymbol{C}}$ by $\widehat{C}_{iv} = \sum_{m,n=1}^{d} U_{im} S_{mn} V_{vn}^{*}$ where *d*<8. Then, we solved the least square problem with $\widehat{\boldsymbol{C}}$. We determined the optimal *d* by the leave-one-out cross-validation scheme: One of the eight bins was excluded in evaluating $\hat{\boldsymbol{v}}$ and then we computed the "test error" by computing $E(\hat{\boldsymbol{v}})$ only with the held-out data. We found that the average test error was minimal at *d* = 3, and used this for final results. For each case, we computed the jackknife mean and standard error of the mean predictor by using the leave-one-out procedure again.

Computational model. Each cortical area is constituted by two cortical layers (or more generally, laminar modules) that describe the dynamics of superficial and deep layers. A laminar module contains one

excitatory and one inhibitory population, and the dynamics of their respective firing rates $r_E(t)$ and $r_I(t)$ are described by the following equations:

$$\tau_E \frac{dr_E(t)}{dt} = -r_E(t) + F(I_E) + \sqrt{\tau_E}\,\sigma\,\xi(t)$$

$$\tau_I \frac{dr_I(t)}{dt} = -r_I(t) + F(I_I) + \sqrt{\tau_I}\,\sigma\,\xi(t)$$

Here, τ_E, τ_I denote the time scales for the excitatory and inhibitory populations respectively, and $\xi_E(t), \xi_I(t)$ are Gaussian white noise terms of zero mean and standard deviation σ. For superficial layers, we choose $\tau_E = 6\,ms, \tau_I = 15\,ms$ and $\sigma = 0.3$, which leads to a noisy oscillatory dynamics in the gamma range, and for deep layers we choose $\tau_E = 48\,ms, \tau_I = 120\,ms$ and $\sigma = 0.45$, which leads to noisy oscillations in the theta and low alpha range. Note that the relatively high values for the time constants in deep layers are thought to reflect other slow biophysical factors not explicitly included in the model, such as the dynamics of NMDA receptors.

The function $F(x) = x/(1 - \exp(-x))$ is the input-output transfer function of each population, which transforms the incoming input currents into their corresponding cell-averaged firing rates. The argument of the transfer function is the incoming current for each population, which involves a background term, a local term and a long-range term. The background term is a default constant current only received by excitatory neurons in S1 and M1, and it is $I_{bg} = 4$ for superficial excitatory neurons and $I_{bg} = 1$ for deep excitatory neurons. The local term involves the input coming from neurons within the area, and it is given by

$$I^E_{local} = 1.5\,r_E - 3.25\,r_I + I^E_{interlaminar}$$

$$I^I_{local} = 3.5\,r_E - 2.5\,r_I + I^I_{interlaminar}$$

Here, the numbers denote the strengths of the synaptic projections considered. The interlaminar terms are contributions from a different layer than the one the population is in. The only interlaminar

projections are from superficial excitatory to deep excitatory neurons, with synaptic strength 1, and from deep excitatory to superficial inhibitory neurons, with synaptic strength 0.75 (11).

Finally, the long-range term includes currents coming from other neocortical or subcortical areas. These currents follow the general form $J_{ab}r_b$, (with J_{ab} being the synaptic strength from area 'b' to area 'a') and therefore we will specify only the synaptic coupling strengths to characterize them.

Following anatomical evidence (12), we consider excitatory projections from superficial S1 neurons to both superficial (strength 0.52) and deep (0.25) excitatory M1 neurons, and from deep S1 neurons to superficial (0.25) and deep (0.23) excitatory M1 neurons. In the opposite direction, we consider excitatory projections from superficial M1 neurons to both superficial (0.3) and deep (0.5) S1 excitatory neurons, and from deep M1 neurons to deep (1) S1 excitatory neurons.

The dynamics of the firing rate of the trigeminal nucleus (TN), the thalamic nuclei (VPM, VL and Pom) and cerebellar populations (PC, CN and ZI) are each described by equations of the type

$$\tau \frac{d\,r(t)}{dt} = -r(t) + f(I)$$

Here, $\tau = 6\ ms$ is the characteristic time constant and the transfer function is a threshold-linear function (i.e. $f(x) = Ax$, with A being the gain of the population, for x>0, and $f(x) = 0$ otherwise). The gain parameter A takes the values 3, 10, 1, 1, 0.5, 5 and 0.2 for areas TN, PC, CN, ZI, VPM, VL and Pom, respectively. Air puffs are modeled as a constant input (max I = 10) to TN, while optogenetic stimulation to PC is modeled as a constant input (I = 1). Cerebellar areas have an inhibitory projection (strength -1) from PC to CN, and an excitatory projection (strength 1) from CN to ZI. In addition, PC and CN received excitatory background currents of 0.1 and 21 respectively, and ZI receives an inhibitory background current of 12 (which can be also interpreted as a high firing threshold). Thalamic nuclei VPM received an excitatory projection (strength 1) from TN, VL receives projections from CN (strength 1) and ZI (strength -3), and Pom receives projections from TN (1), CN (0.2) and ZI (-0.5). Projections from VPM reach superficial excitatory (strength 0.66), deep excitatory (0.13) and inhibitory (0.2) populations of S1. Regarding M1, it receives projections from Pom to all its excitatory (0.33) and inhibitory (0.5) populations, and deep excitatory M1 neurons also receive a

projection (0.6) from VL. When projections from Pom to S1 are considered (see SI Appendix, Fig. S20), they target both excitatory (0.2) and inhibitory (0.15) populations in S1.

To mimic the depth of the recording electrodes for wS1 and wM1 in experiments, we estimate the LFP signal in the model by a weighted average of the excitatory superficial and deep layers, with a superficial:deep ratio of 1:9 for wS1 (i.e. deep layers) and 4:6 for wM1 (as it targets more superficial layers but it would still pick up signals from apical dendrites' layer V neurons).

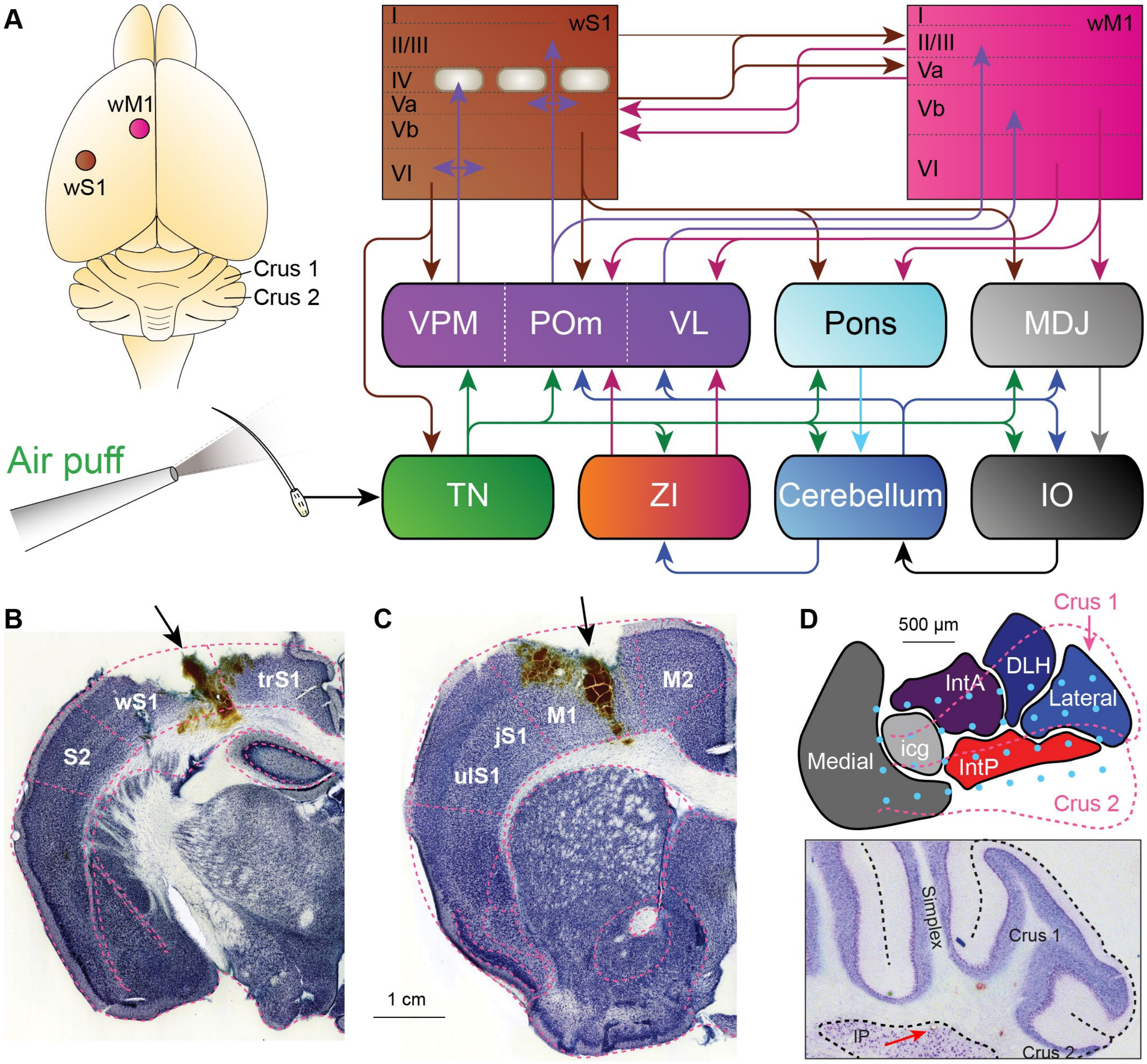

Fig. S1. Interaction of cerebellum and cerebral cortex.

A Simplified anatomical scheme. IO = inferior olive; MDJ = mesodiencephalic junction; Pom = posterior medial nucleus; TN = trigeminal nuclei; VL = ventrolateral nucleus; VPM = ventroposterior medial nucleus; ZI = zona incerta. **B** The electrode position was verified to be in wS1 using post mortem histology. The arrow indicates the location of the electrode (brown markers). Note that the damage to the cortex was inflicted during the removal and preparation of the brain after completion of the experiment. **C** The same for the M1. **D** The recording sites in the cerebellar cortex and cerebellar nuclei were established using a combination of entry points (see a photograph of the craniotomy in Fig. S2G) and electrolytical lesions performed directly after the experiments. In this way we could link the position of electrodes in the matrix (light blue dots) with the cerebellar lobules (dotted magenta lines) and the cerebellar nuclei. In the lower photomicrograph, a lesion in the interposed (IP) nucleus is shown (red arrow).

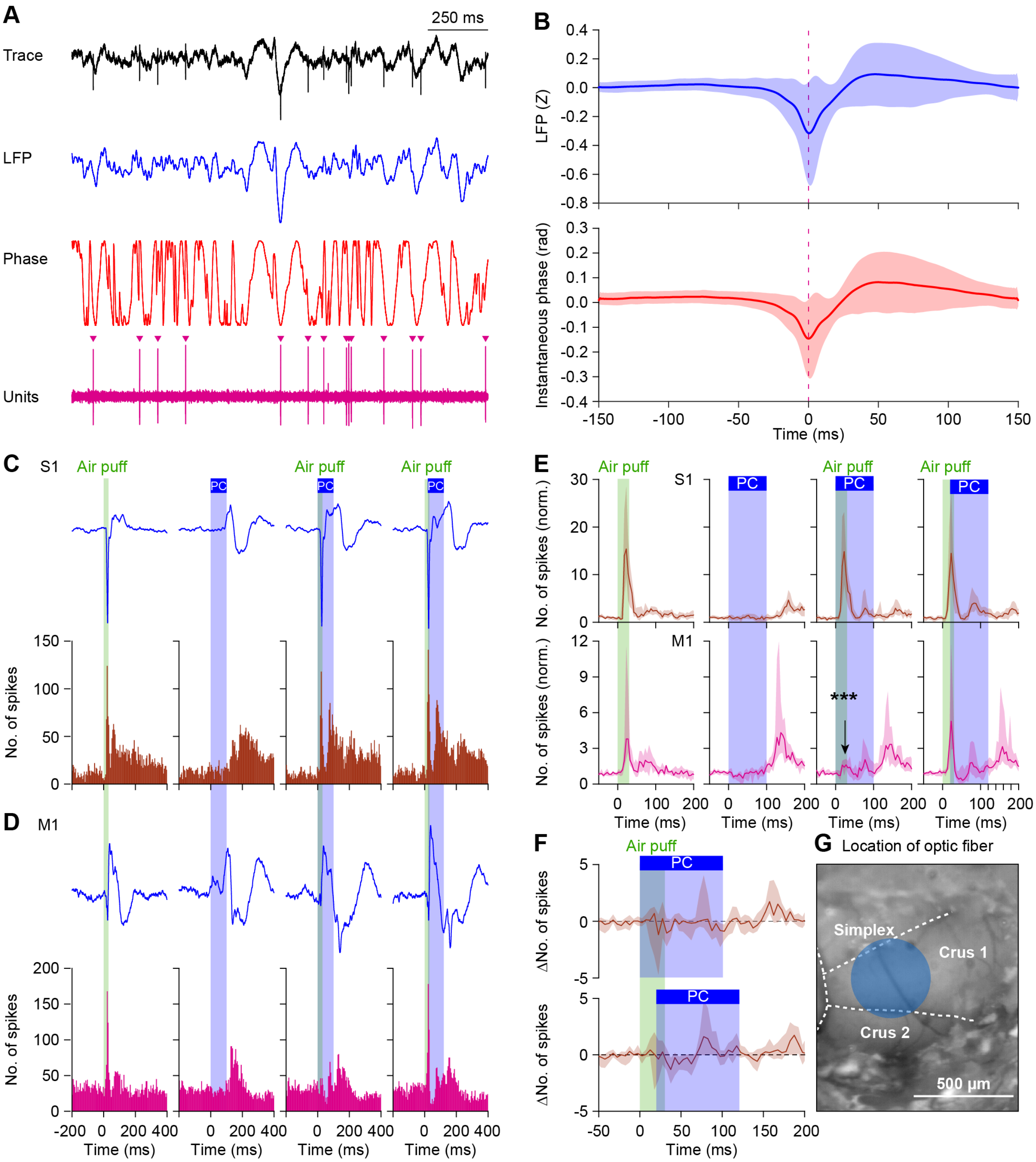

Fig. S2. Multi-unit activity occurs preferentially during the negative phase of the LFP signal
A Exemplary recording of electrode no. 9 in wS1 (see Fig. 1B) during a session without stimulation. From top to bottom, the traces show the recording without post-hoc filtering, with a 80 Hz low-pass filter revealing the local field potential (LFP), instantaneous phase transform of the LFP signal, and with a 1 kHz high-pass filter visualizing the multi-unit activity. The triangles mark the detected multi-unit activity (spikes). Note that most spikes occurred during the negative phase of the LFP. **B** Spike-triggered averages of the LFP signal (top) and instantaneous phase (bottom) based on >1 million spikes recorded in four animals, irrespective of the recording location. Both representations confirm the preferential occurrence of spikes during the negative phase of the LFP. Shaded areas indicate sd. **C** The averaged LFP signals (top) and the peri-stimulus histograms (below) of multi-unit activity from electrode 4 during a representative recording in wS1.

The four stimulation conditions were, from left to right, air puff stimulation of the whiskers, optogenetic stimulation of the Purkinje cells, both stimuli combined simultaneously, and with a lag of 20 ms between sensory and Purkinje cell stimulation. **D** The same for electrode 6 in wM1. **E** Averaged peri-stimulus time histograms of the four stimulus conditions of all multi-unit recordings in wS1 (n = 22) and wM1 (n = 13). The impact of optogenetic stimulation on spiking was stronger in wM1 than in wS1. The most striking impact was the strong reduction of the initial peak in wM1 activity following whisker pad stimulation by simultaneous optogenetic Purkinje cell stimulation (black arrow). This effect was canceled by a delay of 20 ms of the Purkinje cell stimulation (time interval: 10-20 ms after air puff stimulation; $p < 0.001$, $F = 21.385$, df = 2, Friedmann's ANOVA, post-hoc comparisons relative to air puff alone: air puff + Purkinje cell stimulation: $p < 0.001$, $F = 1.769$; air puff + delayed Purkinje cell stimulation: $p = 0.170$, $F = 0.538$). During the later phase (85-100 ms) of Purkinje cell stimulation, decreased multi-unit activity was observed when both stimuli were combined simultaneously. **F** The latter effect is illustrated by the difference of the response during simultaneous (top) and time-delayed (bottom) combined stimulation and the air puff stimulation: $p = 0.036$, W = -112 and $p = 0.946$, W = -5, respectively, Wilcoxon matched pairs tests). **G** Photograph of a craniotomy in which the approximate location of the optic fiber (400 μm diameter and delivering 7 mW of blue light). Optogenetic stimulation in Figs. 1-3 and S2-19 was delivered using this configuration.

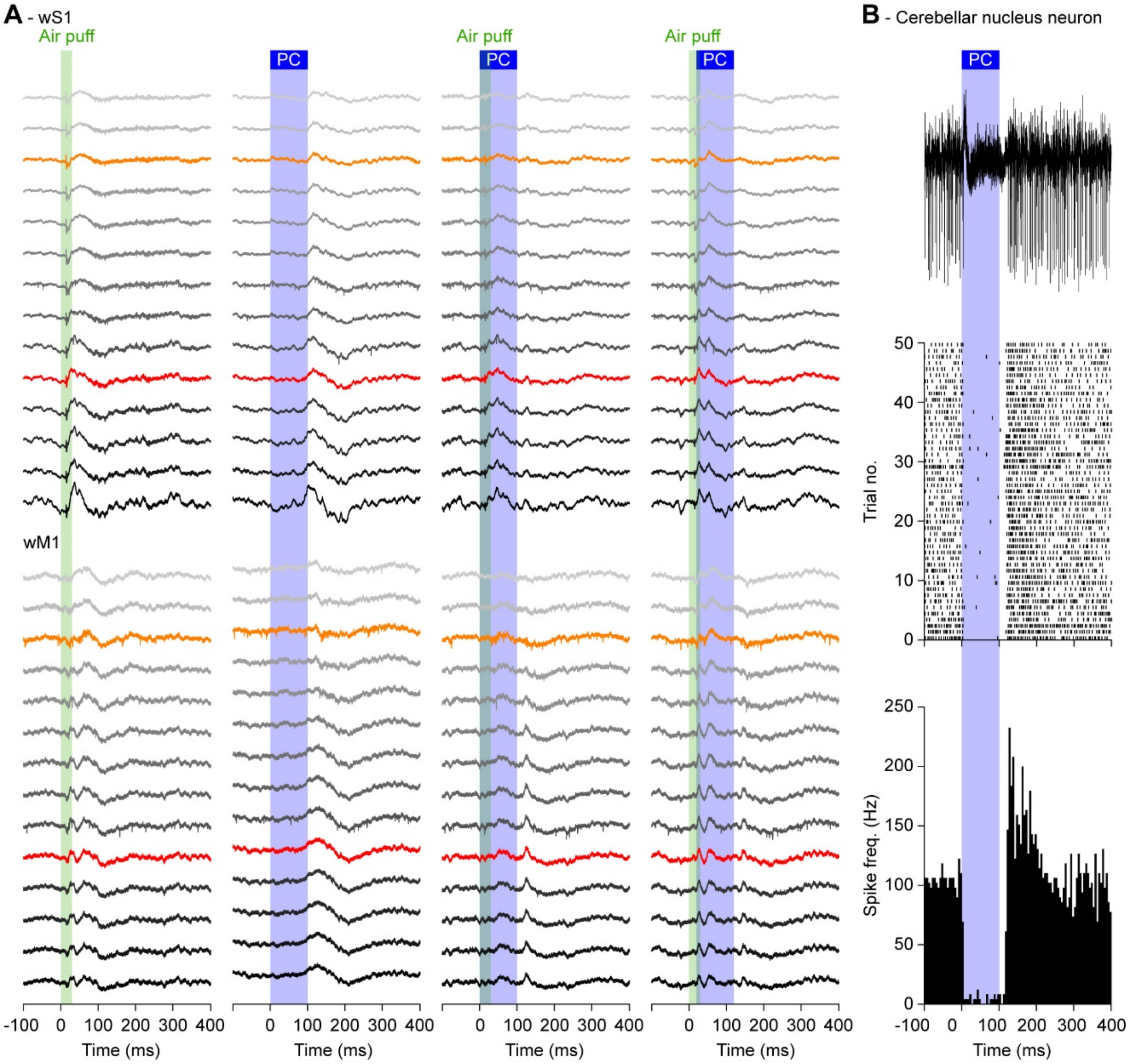

Fig. S3. Purkinje cell stimulation reduces the impact of whisker stimulation on wS1 and wM1.
A Air puff stimulation of the large facial whiskers evoked sensory responses in contralateral wS1 and wM1, recorded here as deviations in the local field potential (LFP) of a randomly selected single trial (left column). The LFP recordings were made using linear silicon probes with 100 µm inter-electrode distances. The recordings are organized from superficial to deep (color code as in Fig. 1B). Electrodes 3 and 10, that were used for most analyses in this study, are marked with orange and red, respectively. Optogenetic stimulation of Purkinje cells (PC) was done with an optic fiber with a diameter of 400 µm placed on the center of crus 1 (see Fig. S2G), leading to a delayed response in both wS1 and wM1 (2nd column). The other columns depict randomly selected trials from the same experiment, showing respectively the combined sensory and optogenetic Purkinje cell stimulation and the latter with a delay of 20 ms before the onset of the Purkinje cell stimulation. **B** Optogenetic Purkinje cell stimulation leads to a pause in firing of an exemplary neuron in the cerebellar nuclei, followed by rebound firing after the end of stimulation. Further analysis of cerebellar nuclear activity is presented in Fig. S20.

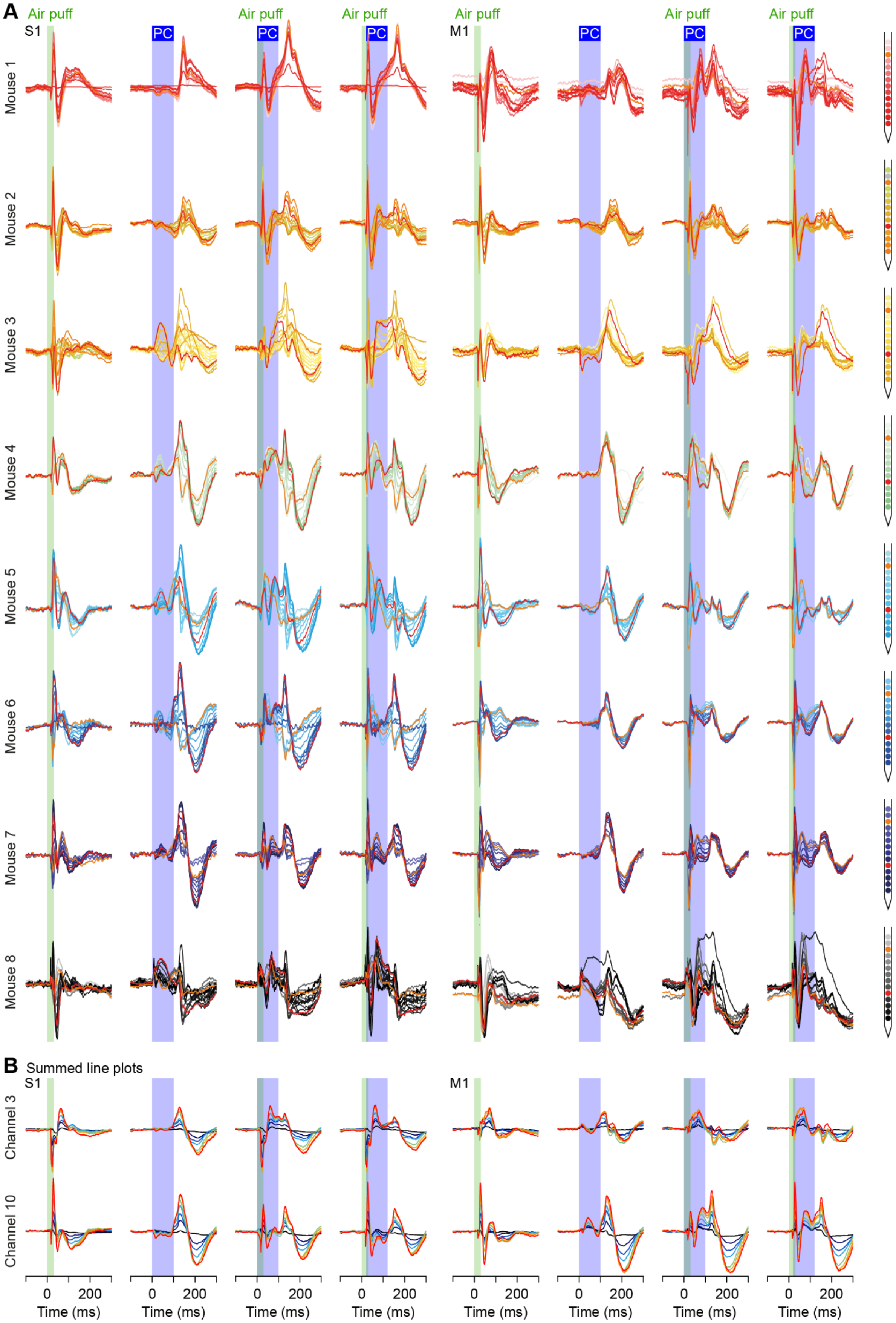

A
Air puff
S1
PC
M1
Mouse 1
Mouse 2
Mouse 3
Mouse 4
Mouse 5
Mouse 6
Mouse 7
Mouse 8
B
Summed line plots
Channel 3
Channel 10
0
200
Time (ms)

Figure S4. LFP recordings of individual mice share similar features.
A Each row shows the averaged traces during the different stimulus conditions recorded in wS1 (left) and wM1 (right). Each color indicates a different electrode, with brighter colors corresponding to the more superficial and darker colors to the deeper channels, according to the schematics on the far right. Channels 3 and 10 are always indicated in orange and red, respectively. **B** Summed line plots of the channels 3 and 10 of each mouse normalized so that the red line indicates the average. Each color symbolizes one mouse, consistent with the color coding in **A**. This shows that all mice contributed to the different phases of the LPF responses. Air puffs were applied to the contralateral large facial whiskers and optogenetic stimulation was delivered with a 400 μm optic fiber placed centrally on the surface of crus 1 (see Fig. S2G) using a power of 7 mW.

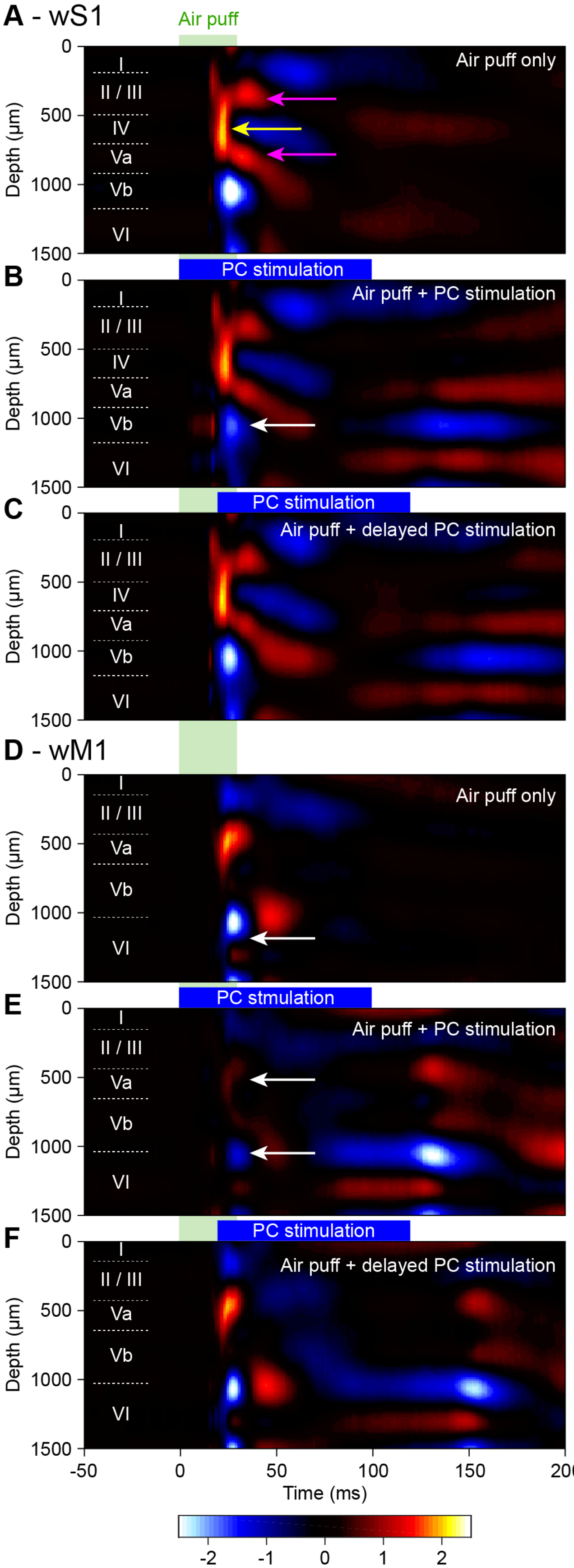

Fig. S5. Purkinje cell stimulation reduces sensory responses in wS1 and wM1.
Current source density analysis of the averaged local field potentials across the layers of the whisker area of S1 (see Fig. 1E-J) during whisker air puff stimulation alone (**A**) or in combination with optogenetic stimulation of Purkinje cells (**B**) using an optic fiber with a diameter of 400 µm placed on the center of crus 1 (see Fig. S2G). Purkinje cell stimulation applied simultaneously with whisker stimulation suppressed predominantly the fast current sinks (blue; white arrow), but a 20 ms delay between air puff and whisker stimulation (**C**) largely restores the impact of air puff stimulation. The colored arrows are referred to in the main text. **D-F** The same was true for the whisker area of wM1. The heat maps indicate the averaged values of 8 mice with color scaling in arbitrary units. The layers of wS1 and wM1 are indicated by approximation.

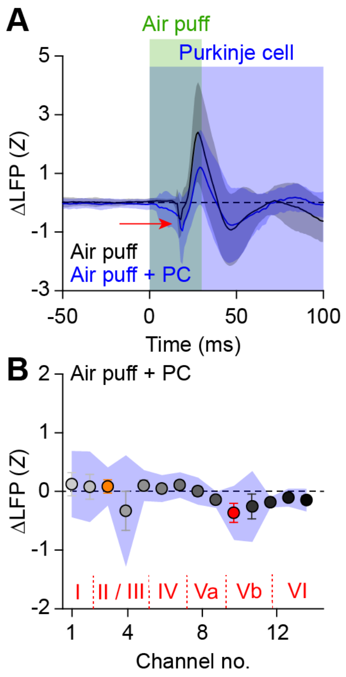

Fig. S6. Optogenetic Purkinje cell stimulation does not affect the initial sensory response in wS1. **A** Air puff whisker stimulation triggers a fast negative peak in LFP signal on channel 10 in the subgranular layer of wS1 (see red arrow). This negative peak is enhanced by simultaneous optogenetic stimulation of Purkinje cells using a 400 µm diameter optic fiber (P = 7 mW) placed centrally on the surface of crus 1 (see Fig. S2G). Traces are the averaged *Z* scored LFP responses of 8 mice. Shades indicate sd. **B** The impact of simultaneous Purkinje cell stimulation on the initial negative LPF peak following whisker stimulation was not statistically significant. Error bars indicate SEM and shaded area sd. *n* = 100 trials each in *N* = 8 mice. See Table S1 for statistical details.

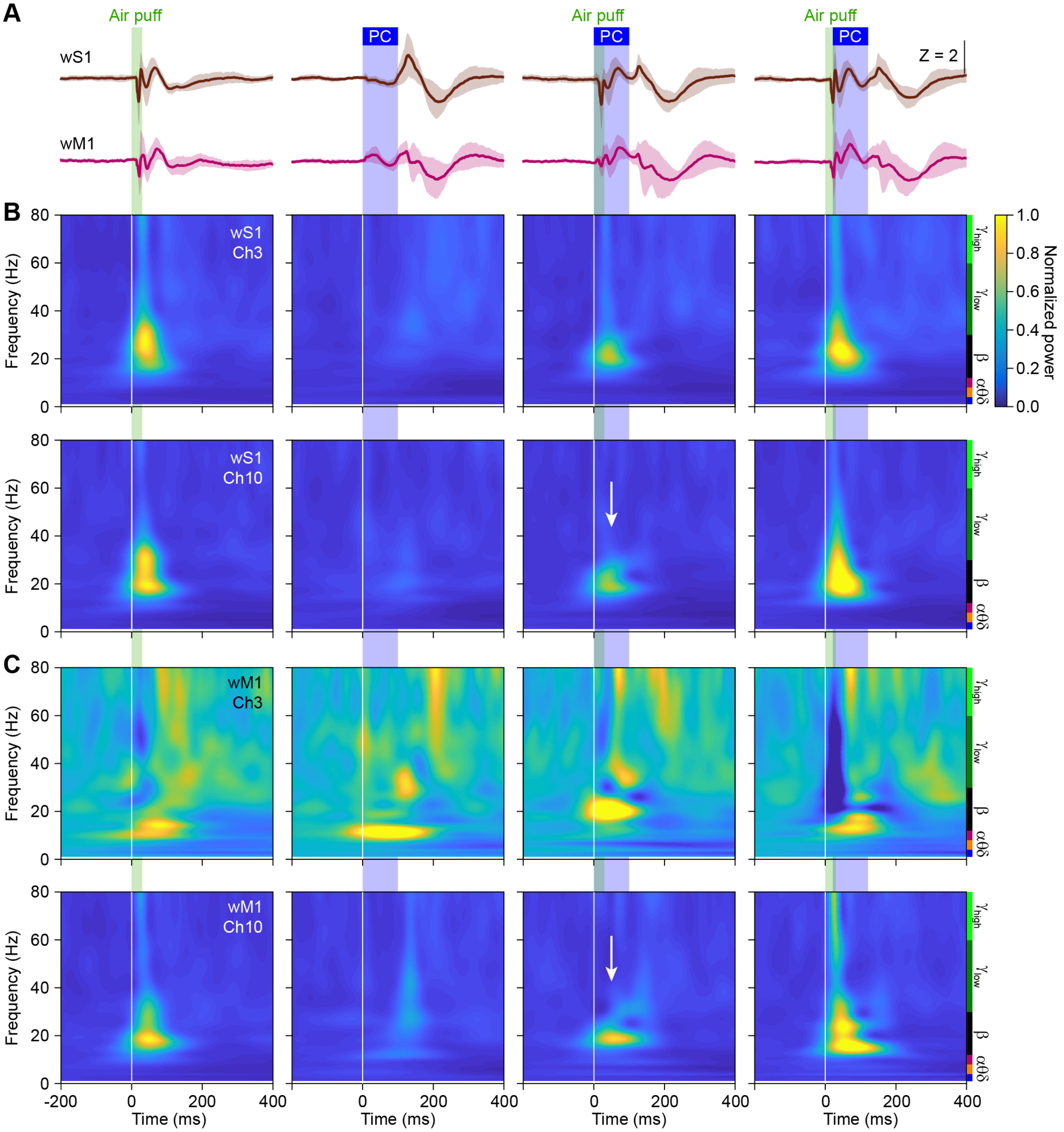

Fig. S7. LFP power spectra reveal suppression of sensory-induced gamma band activity by simultaneous optogenetic Purkinje cell stimulation.

A Averaged LFP signals in subgranular wS1 and supragranular wM1 following either air puff stimulation of the contralateral facial whiskers, optogenetic Purkinje cell (PC) stimulation, or a combination of both. In the column on the right, there was a 20 ms delay between the onset of air puff and Purkinje cell stimulation. Purkinje cell stimulation was performed with an optic fiber with a 400 μm diameter placed on the center of crus 1. These traces are copied from Fig. 2A. **B** Power spectra of channels 3 (top) and 10 (bottom) placed respectively in the supra- and subgranular layers of wS1. The power is normalized by dividing it by a baseline power spectrum (average power spectrum well before (~100 ms) any stimulation). **C** The same analysis applied to channels 3 and 10 of wM1. Data in **B** and **C** originate from 100 trials per condition in a representative mouse.

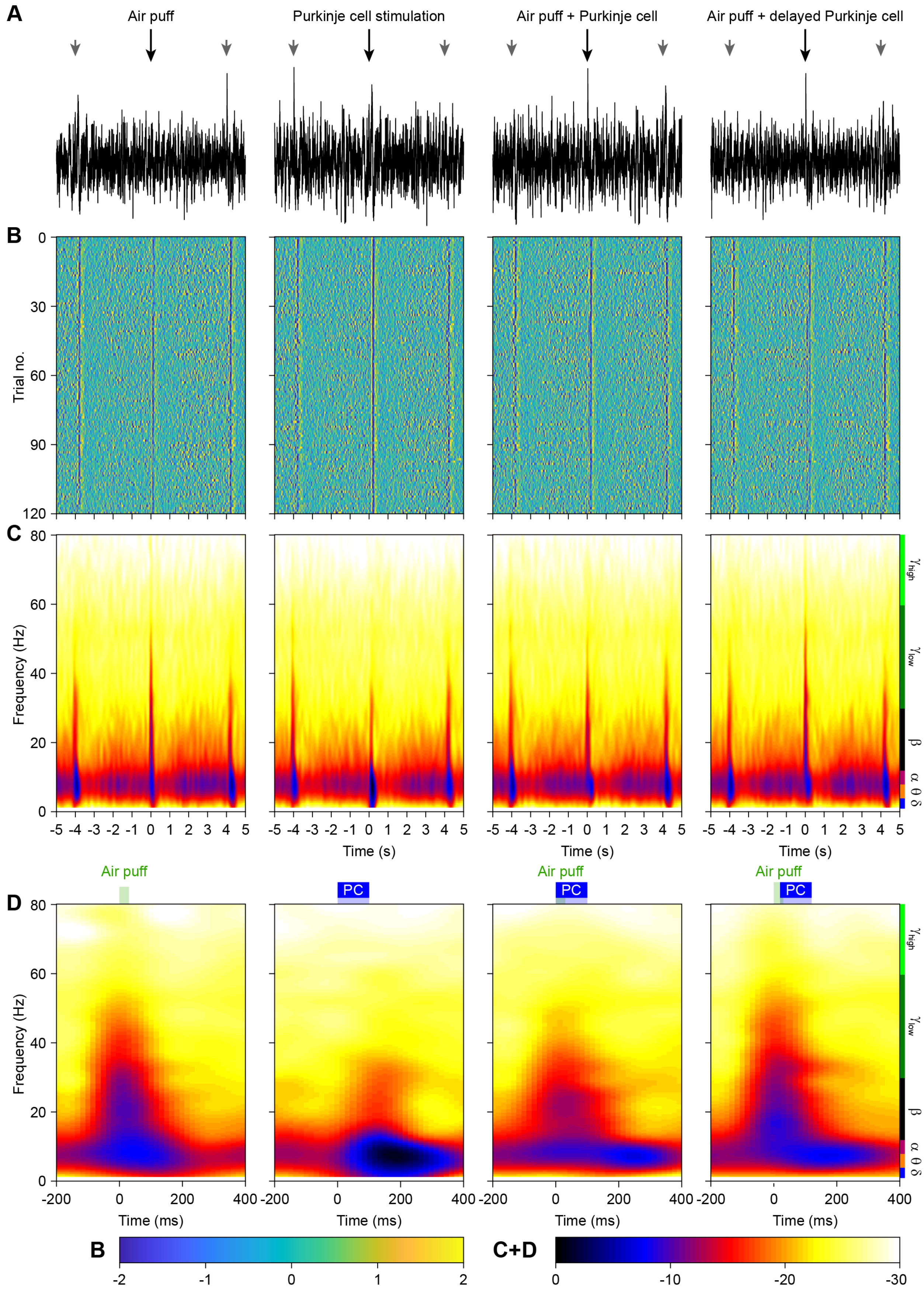
A
Air puff
Purkinje cell stimulation
Air puff + Purkinje cell
Air puff + delayed Purkinje cell
B
Trial no.
C
Frequency (Hz)
Time (s)
D
Air puff
PC
Time (ms)
B
C+D

Figure S8 – Spectral analysis of the subgranular layers of wS1.
A Single trial LFP data. Black arrows (middle) indicate the stimulus, the side arrows indicate the previous and consequent stimuli. Since the stimulus sequence was randomized, the side arrows represent mixtures of different types of stimuli. **B** Color coded LFP traces, displaying the Z score. Each row corresponds to a single trial. **C** Average spectrograms for snippets around stimuli at the same time scale as **A** and **B**. **D** Data from **C**, zoomed in around stimulus. Complex oscillatory behavior is observed at rest across most bands and is potentiated during evoked responses. All data are derived from channel 10 (placed in the subgranular layer) in wS1 and originate from a representative experiment.

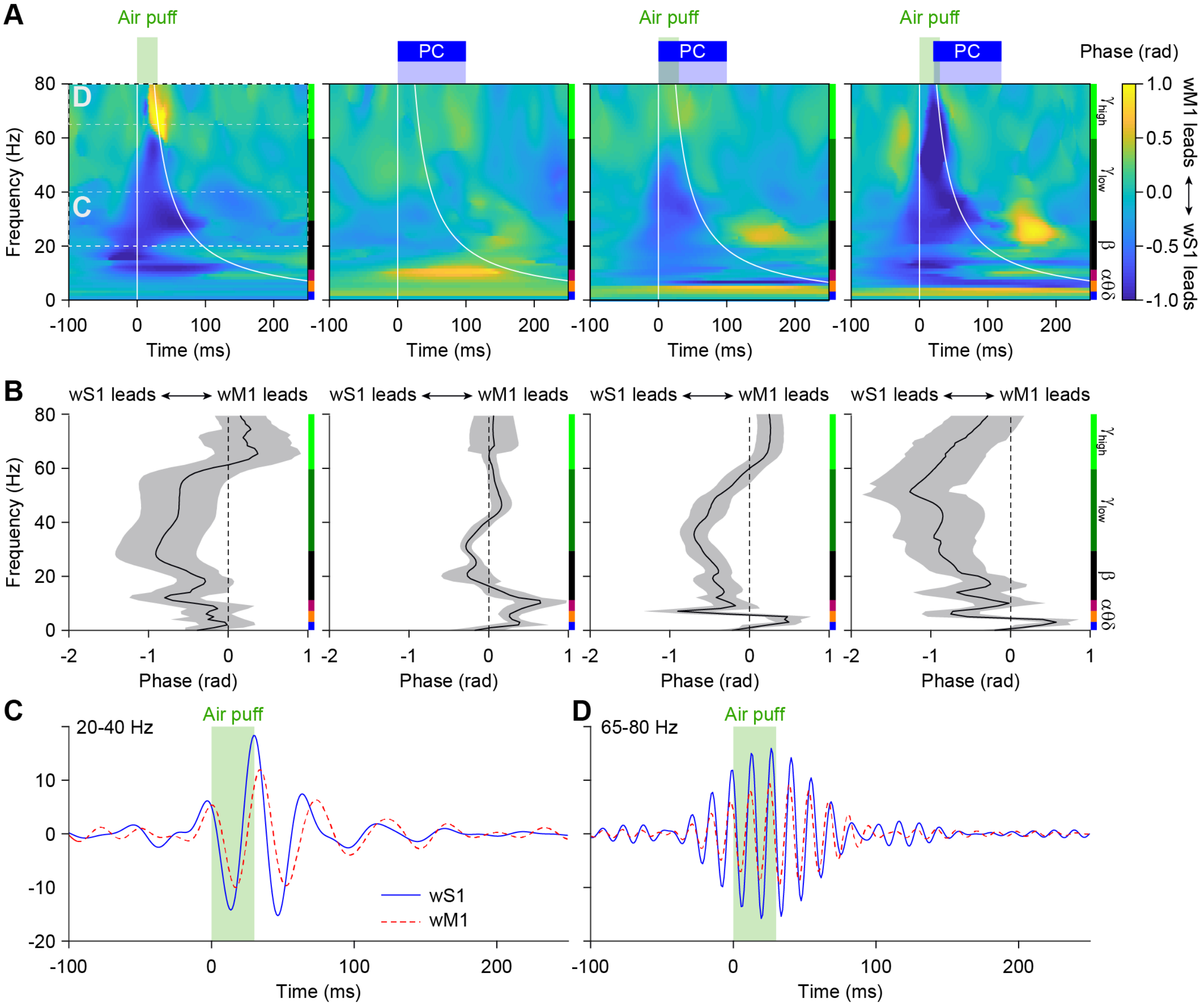

Figure S9 – wS1 often leads wM1.
A Heat maps illustrating the phase differences between the subgranular layer of wS1 (electrode 10) and the supragranular layer of wM1 (electrode 3) during the four different stimulus conditions: air puff to the whiskers optogenetic stimulation of Purkinje cells, both stimuli combined simultaneously and with a 20 ms delay. Optogenetic stimulation was delivered by an optic fiber with a diameter of 400 µm placed centrally on crus 1 (see Fig. S2G). Negative values (blue colors) indicate a phase lead of wM1, positive values (yellow colors) a phase lead of wS1. **B** Averaged phase difference (*N* = 8 mice). Shaded areas indicate SEM. **C** Over most frequencies, wS1 led wM1, as illustrated here for the LFP signal after band-pass filtering at 20-40 Hz. **D** Only at higher gamma frequencies, wM1 led wS1 (65-80 Hz band-pass filter).

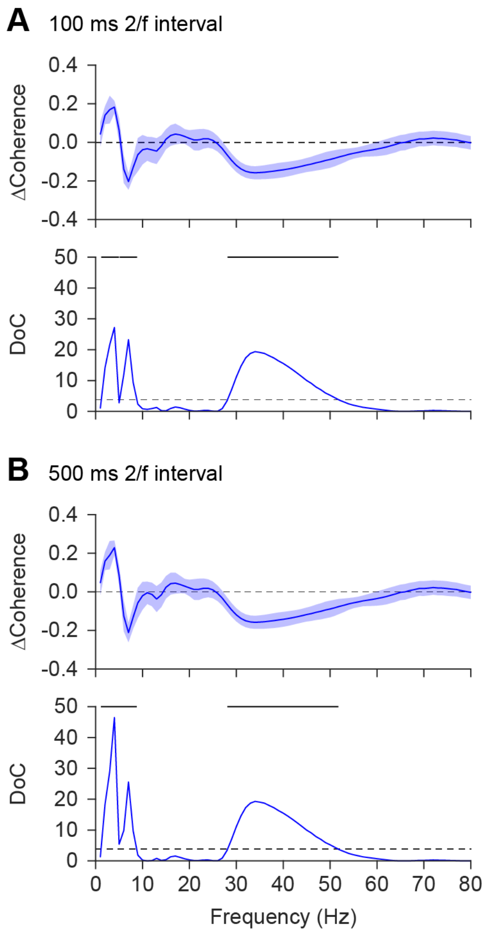

Figure S10 – Difference of Coherence (DoC) analysis.

A Using the frequency dependent time window described by 2*(1/f) results in relatively large time windows in which the lower frequency coherence is averaged. To assess the effect of these large windows, we reanalyzed the difference of coherence (DoC, see Methods) using different time limits. Top: difference of the coherence between trials with only air puff stimulation and those with combined air puff and Purkinje cell stimulation. Bottom: DoC analysis using a maximum window of 100 ms. This results in a 100 ms window for the frequencies of 1-20 Hz, and a frequency dependent window for all higher frequencies. Significant frequencies are indicated with the horizontal lines. **B** As **A**, but with a maximum window of 500 ms. Here the frequencies 1-4 Hz are averaged over 500 ms, and all higher frequencies use the frequency dependent window. Shaded areas indicate SEM.

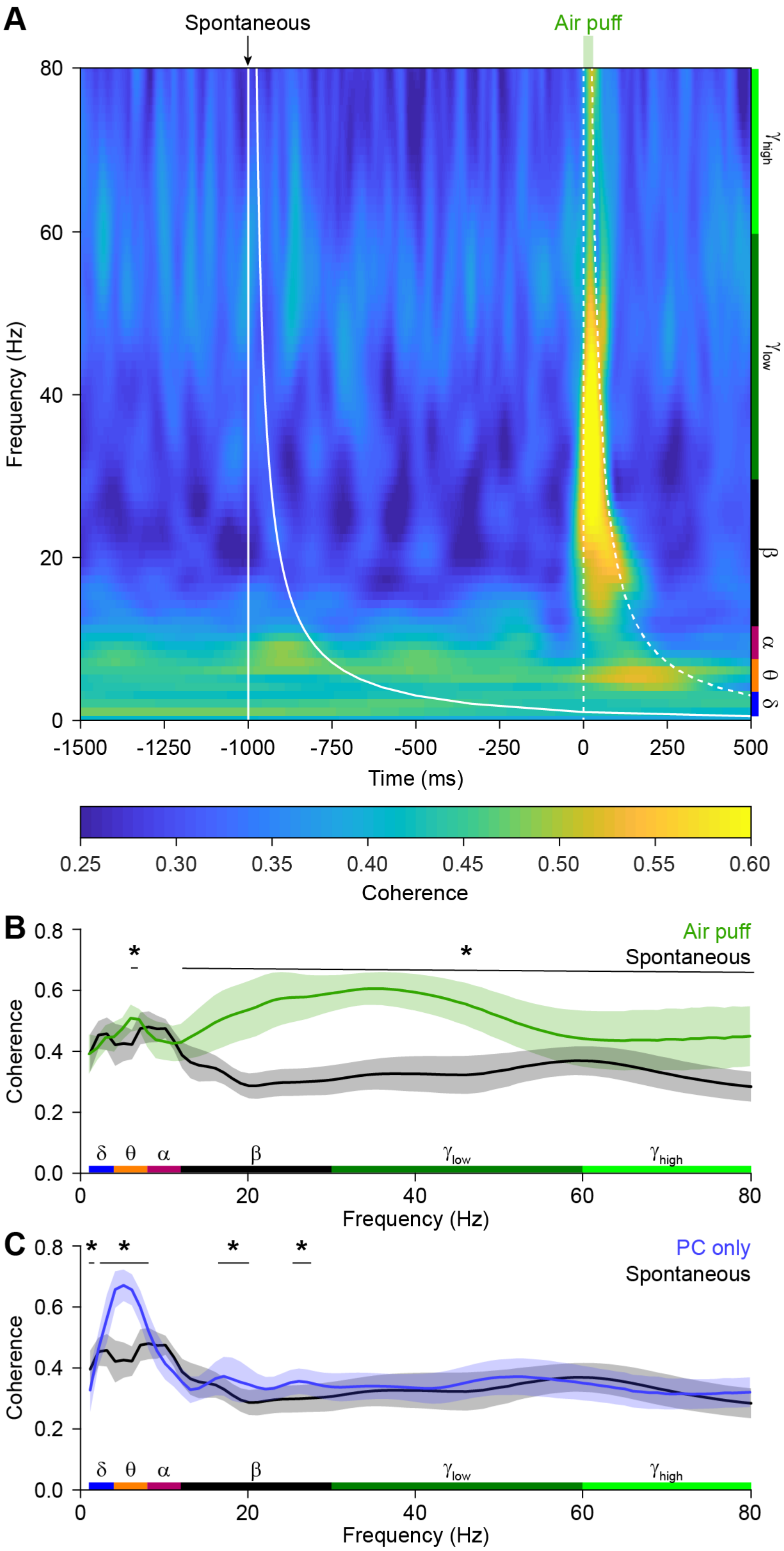

Figure S11. Sensory stimulation increases coherence between wS1 and wM1 in the delta, beta and gamma bands.
A Heat map of the coherence between subgranular wS1 and supragranular wM1 during trials with air puff stimulation of the contralateral whiskers, comparing the inter-trial interval (triggered at -1000 ms) and the sensory response (at 0 ms). Averaged values of 8 mice with 100 trials per mouse. **B** A comparison of the coherence between the inter-trial and trial intervals revealed that air puff stimulation increased the coherence in the theta, beta and gamma bands, but not in the delta and alpha bands. **C** Optogenetic Purkinje cell stimulation, using an optic fiber with a diameter of 400 µm and P = 7 mW placed centrally on the surface of crus 1 (see Fig. S2G), increased the coherence around the theta band relative to inter-trial (spontaneous) coherence. * $p < 0.05$, DoC test.

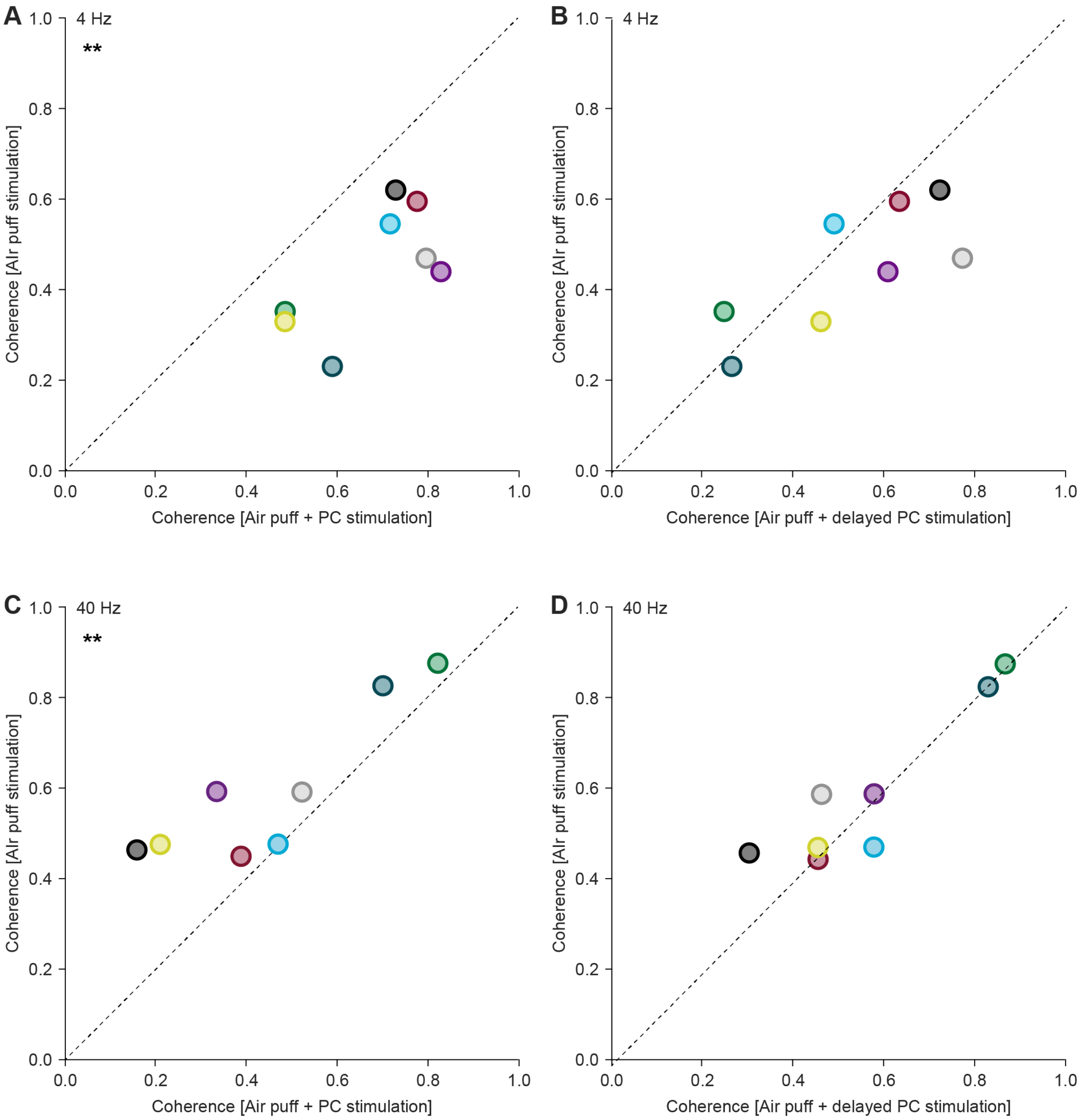

Figure S12. Differential modulation of coherence bands by Purkinje cell stimulation.
Scatter plots comparing the mean wS1-wM1 coherence following air puff stimulation to the whiskers (vertical axis) and during combined air puff and optogenetic Purkinje cell stimulation (horizontal axis). The mean coherence value was estimated from the frequency-specific window (e.g., see white lines in Fig. 2B). Each symbol corresponds to an individual mouse. Symbols above the 45° line indicate a suppression and those below an increase of sensory-induced coherence. Impact of simultaneous Purkinje cell stimulation on 4 Hz (**A**) and 40 Hz (**C**) coherence. **C** and **D** The same for 20 ms delayed Purkinje cell stimulation. ** $p < 0.010$
4 Hz: $p = 0.002$, $\chi^2 = 13.000$, df = 2, Friedman's two-way ANOVA, with air puff vs. air puff + simultaneous Purkinje cell stimulation (**A**): $p = 0.001$, $\chi^2 = -3.500$; and air puff vs. air puff + 20 ms delayed Purkinje cell stimulation (**B**): $p = 0.953$, $\chi^2 = -1.000$.
40 Hz: $p = 0.008$, $\chi^2 = 9.750$, df = 2, Friedman's two-way ANOVA, with air puff vs. air puff + simultaneous Purkinje cell stimulation (**C**): $p = 0.008$, $\chi^2 = 3.000$; and air puff vs. air puff + 20 ms delayed Purkinje cell stimulation (**D**): $p = 0.073$, $\chi^2 = -2.250$.

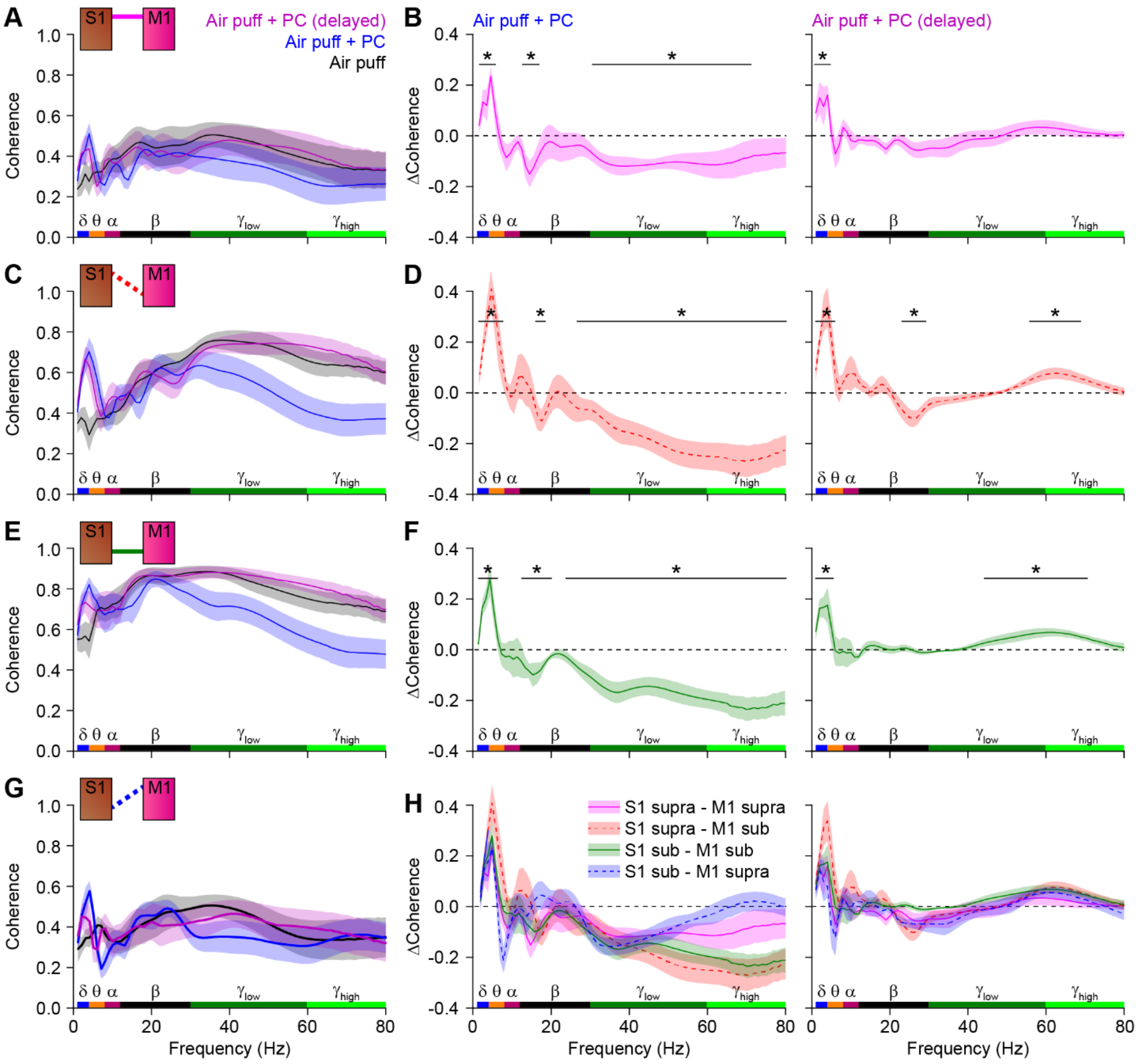

Fig. S13. Cerebellar Purkinje cell stimulation suppresses sensory-induced gamma band coherence between wS1 and wM1.

A Averaged coherence between the supragranular layers of wS1 and wM1 following air puff stimulation of the contralateral facial whiskers in isolation or in combination with simultaneous or 20 ms delayed optogenetic stimulation of Purkinje cells using an optic fiber with a diameter of 400 µm placed on the center of crus 1 (see Fig. S2G). Shaded areas indicate SEM. n = 100 trials per condition each in N = 8 mice. **B** Purkinje cell stimulation suppressed mainly the gamma band coherence induced by air puff sensory stimulation. This effect was largely abolished by introducing a 20 ms delay between the start of the sensory stimulation and that of the Purkinje cells. **C**-**H** The same for the coherence between different layers of wS1 and wM1 as indicated schematically in the upper left corners. Although the details varied to some extent, in all cases Purkinje cell stimulation suppressed sensory-induced gamma band coherence between wS1 and wM1. * $p < 0.05$ ($\chi^2 > 3.84$; difference of coherence test, see Methods).

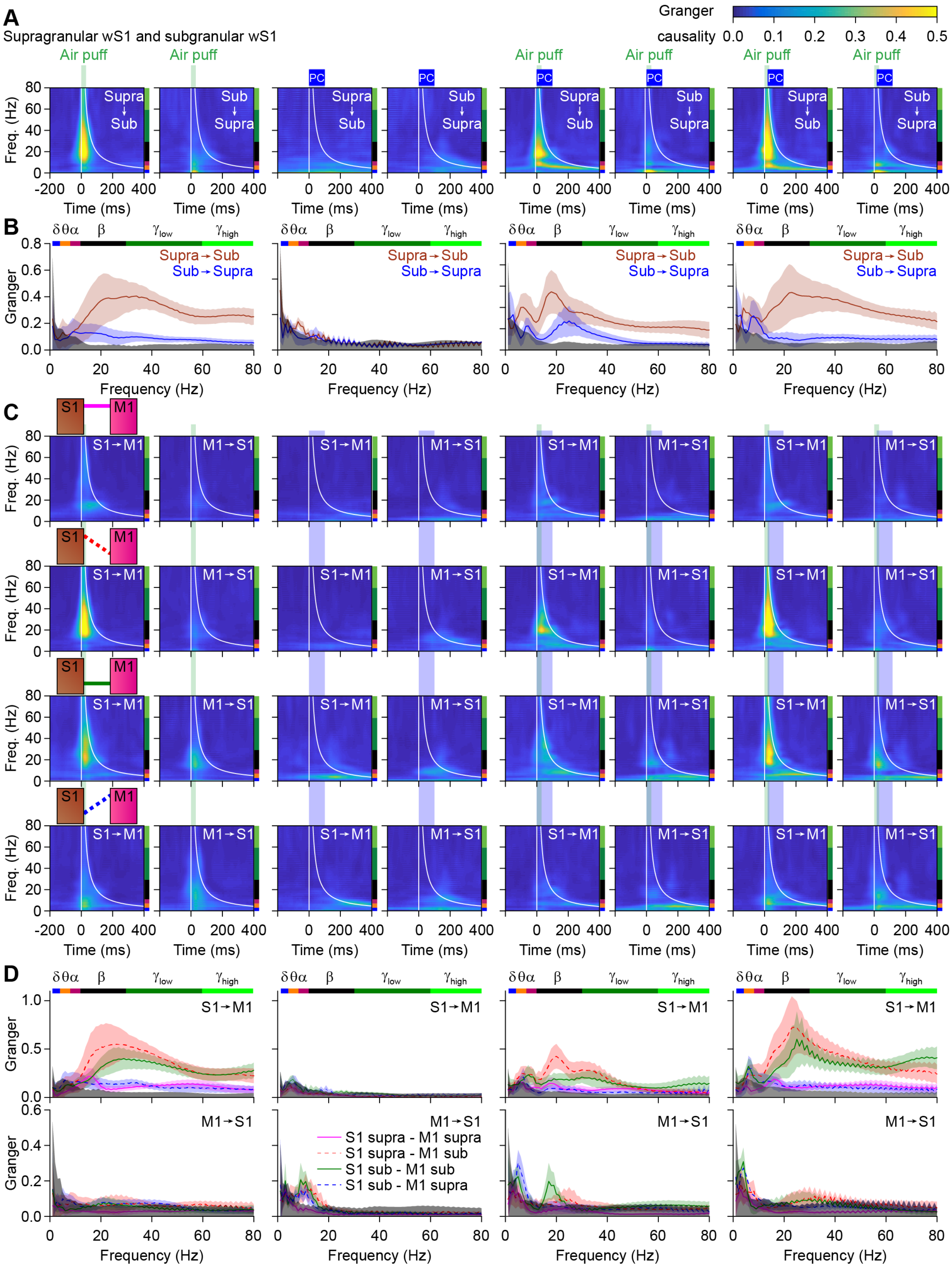
A
Supragranular wS1 and subgranular wS1
Granger causality
Air puff
PC
Supra → Sub
Sub → Supra
Freq. (Hz)
Time (ms)
B
Granger
Frequency (Hz)
C
S1
M1
S1→M1
M1→S1
D
S1 supra - M1 supra
S1 supra - M1 sub
S1 sub - M1 sub
S1 sub - M1 supra

Fig. S14. Granger causality analysis.

A Granger causality analysis revealed that the sensory-induced beta and gamma band coherence within wS1 was mainly caused by activity in the supragranular layers. The flow from the superficial to the deeper layers in the gamma, but not the beta, band was disrupted by simultaneous Purkinje cell stimulation. **B** The mean Granger causality values for the different conditions, comparing the flow from superficial to deep layers vs. the flow from deep to superficial layers. Shaded areas around the averages indicate sd. The black area represents the values below the average + 3 sd during spontaneous activity. All activity above the black area can be considered statistically significant. **C** Granger causality analysis of the coherence between wS1 and wM1, expanding on the analysis shown in Fig. 2C-D where the subgranular layers of wS1 were compared to the supragranular layers of wM1 (data copied in the fourth row to facilitate comparisons). This analysis suggest that the sensory-induced gamma band coherence is mainly caused by the superficial layers of wS1, and from there spreads over wS1 and wM1. The strongest interconnections are found between subgranular layers of wS1 and the supragranular layers of wM1 (fourth row). Also here, wS1 drives wM1 stronger than vice versa, but also wM1 has a share in this coherence, stressing the importance of the connections between wS1 and wM1. **D** The mean Granger causality values for the different relations between wS1 and wM1. Shaded areas around the averages indicate sd. The black area represents the values below the average + 3 sd during spontaneous activity. All activity above the black area can be considered statistically significant.

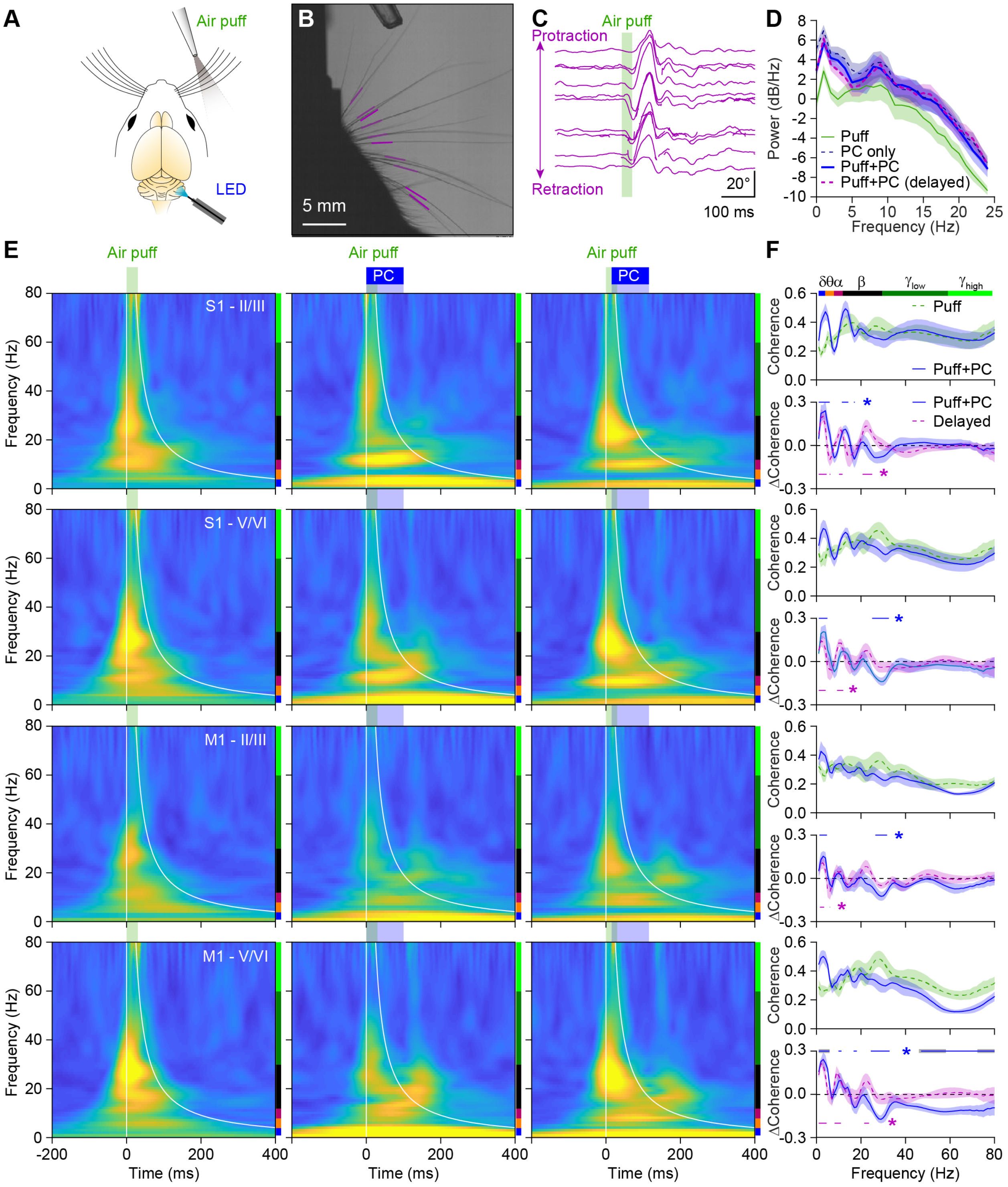

Fig. S15. Cortical coherence during sensory-induced whisker protraction and Purkinje cell stimulation.

A Experimental scheme. **B** The movements of the whiskers were tracked using video-analysis. The colored line fragments indicate the tracked part of the whiskers. **C** Raw output of the whisker tracking algorithm, showing for one trial how an air puff blew the whiskers backwards, after which an active protraction followed. **D** Power spectral density estimates of evoked whisker movements during the different stimulation conditions with and without Purkinje cell (PC) stimulation. Note that in the conditions with PC activation we observed a higher power in the whisker movements over all frequencies. **E** Spectral coherence between

whisker movement and LFP signals recorded in S1 and M1 during trials with air puff, simultaneous air puff + PC stimulation, and air puff + delayed PC presentations. **F** Whisker-LFP coherence averaged using the frequency dependent time-window ((1/f)*2, indicated with white lines in **E**) and the coherence difference between the three different stimulus conditions in solid blue and dashed purple lines, respectively. The differences between the supra- and subgranular layers in wS1 were not significantly different from each other. However, those in wM1 were; this is indicated by the fat grey lines on top of the bottom panel. Note that the panels in wS1 and wM1 panels in **F** are aligned with those in **E**. Significance in the Δcoherence panels is calculated using DoC analysis (see Methods).

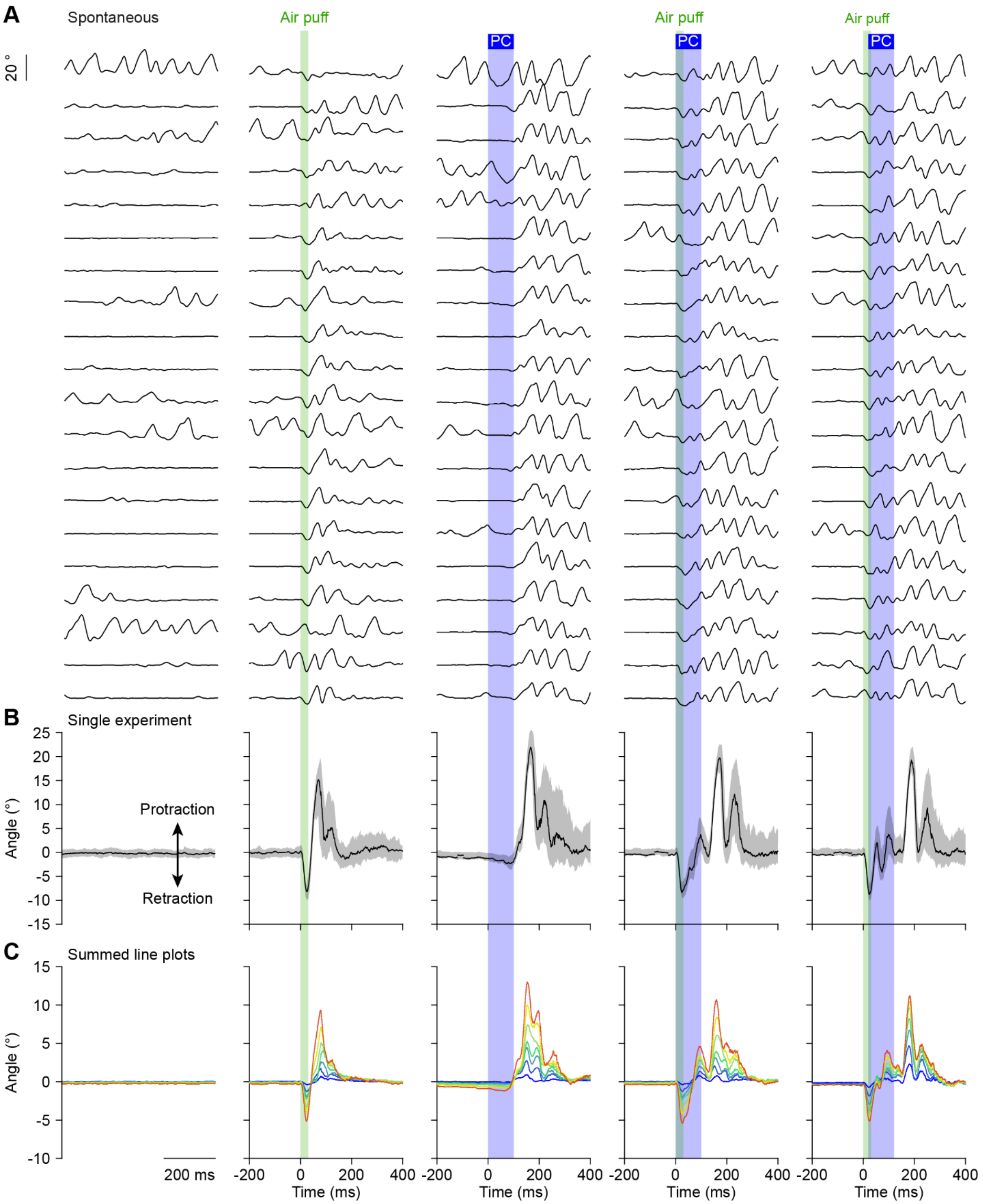

Fig. S16. Stimulus-triggered whisker movements.
A Whisker movements during individual trials during inter-trial intervals (left) and around stimulation. All trials originate from a single experiment. **B** Median whisker movements (shading: IQR) of 100 trials per condition of the experiment illustrated in **A**. **C** Summed line plots of the whisker movements for each stimulus condition. Each line represents one mouse and the traces are normalized so that the red line indicates the population average. Purkinje cell stimulation was done with a 400 μm diameter optic fiber.

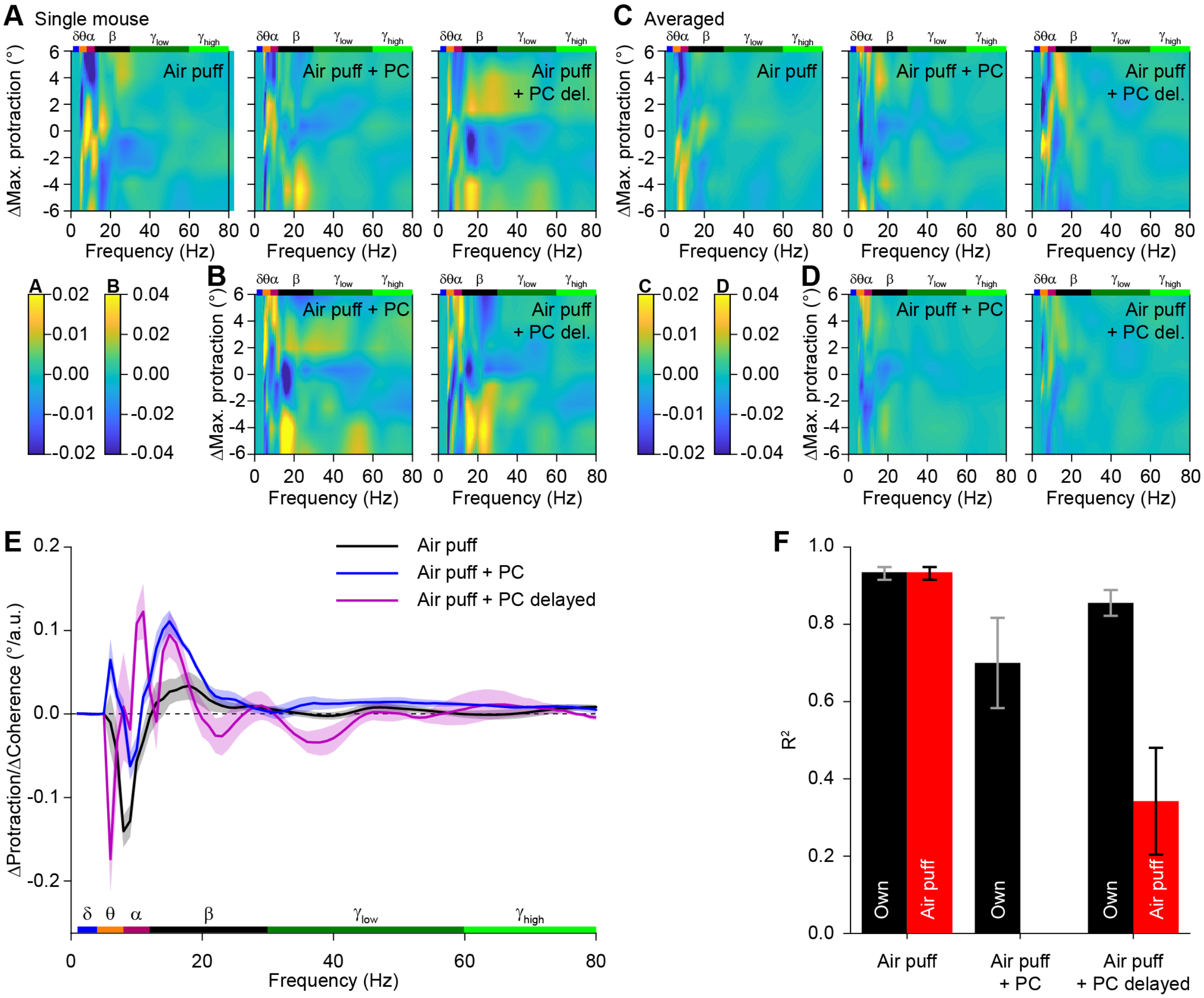

Fig. S17. Coherence encodes the magnitude of the air puff-induced whisker protraction, but it requires the cerebellar output.

A Excess coherence from the mean coherence (ΔCoherence; color) versus maximal whisker protraction from the mean position (ΔMax protraction; y-axis) at each frequency (x-axis), in the air puff-only (left), air puff and synchronous PC activation (middle), and delayed PC activation (right) trials. **B** Difference of ΔCoherence from the air puff-only case with the synchronous (left) and delayed (right) PC activation. In A-B, data is from one animal. **C**, **D** Same as **A** and **B**, averaged across all the animals (*n* = 8). **E** Linear predictor of ΔMax protraction by ΔCoherence, estimated from the data in **C**. Curves and shades are means and SEM, respectively, computed by the jackknife method (see SI Appendix, Supplementary Methods - Regression analysis for coherence and whisker protraction). **F** Goodness of fit (R^2) for the predictors in **E** and data in **C**. Black bars are R^2 when the predictor is used for the data in the same category. Red bars are R^2 when the predictor used is from the air puff-only case (black in **E**). R^2 is shown only when the residual variance is not larger than the original variance, averaged across all the animals (*n* = 8). When their own predictors are used (as for R^2), the mean of the air puff alone paradigm is significantly *higher* than that of the air puff plus PC stimulation with or without delay ($p < 0.001$ for both conditions), and the mean of the air puff plus PC stimulation is *lower* than that of the air puff plus delayed PC stimulation ($p = 0.002$). In addition, the means of all three paradigms, i.e., air puff alone and air puff plus PC stimulation with or without delay, were significantly *higher* than 0 (all $p < 0.001$). Instead, when the air puff predictor is used, the mean of the air puff alone paradigm is *higher* than that of the air puff plus PC with or without delay (both $p < 0.001$). *p* values are derived from *t* tests. See Fig. S18 for the data from individual mice.

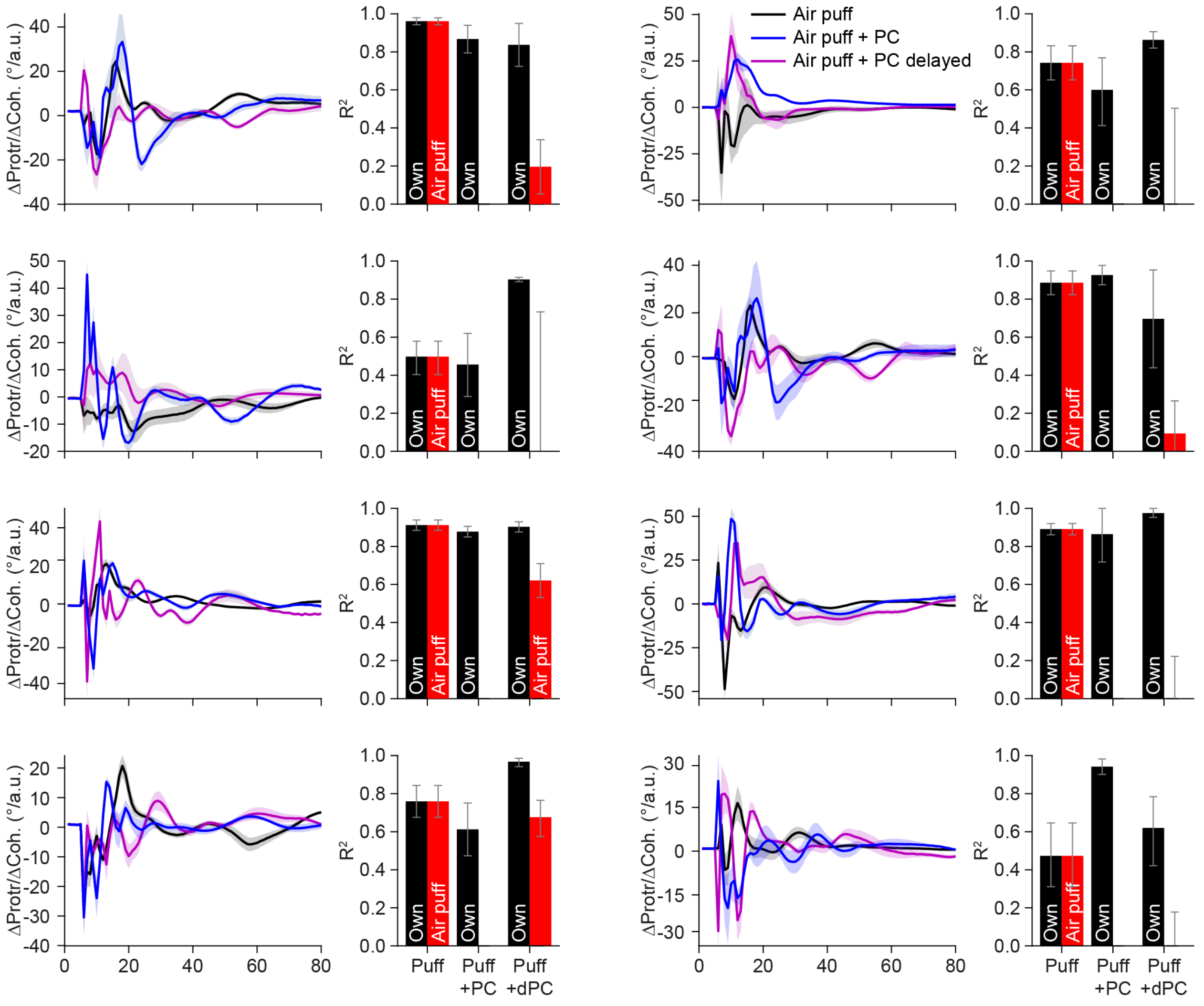

Fig. S18. Coherence encodes the magnitude of the air puff-induced whisker protraction, but it requires the cerebellar output.
Goodness of fit (R^2) as summarized in Fig. S17, shown here for each of the 8 individual mice.
dPC = delayed Purkinje cell stimulation.

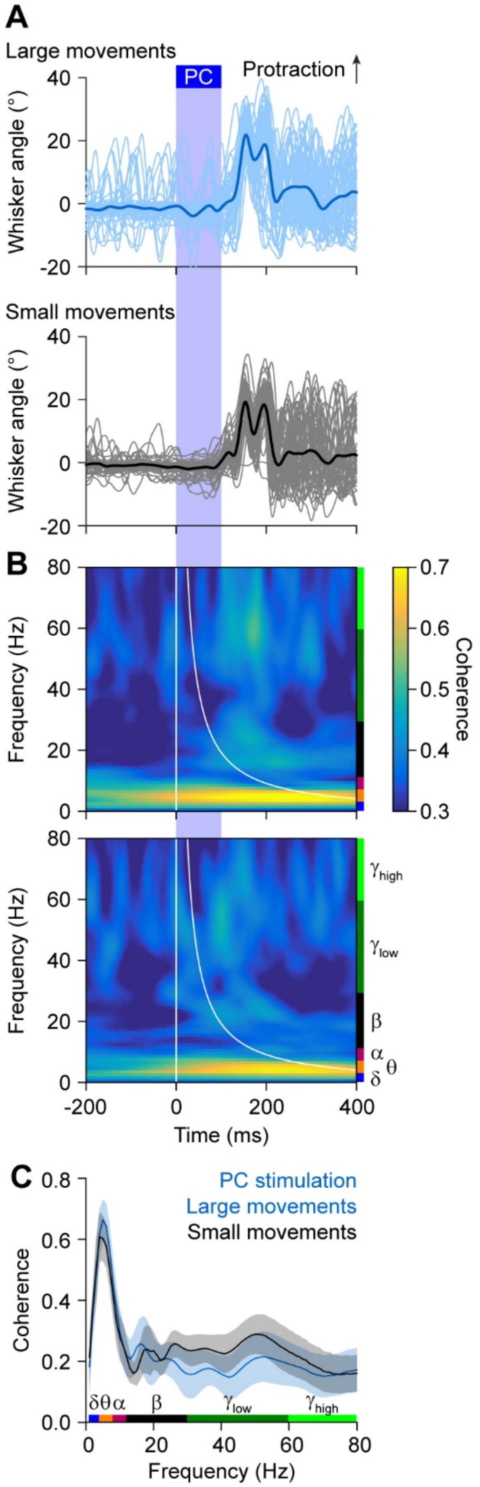

Fig. S19. Optogenetic Purkinje cell stimulation induces delayed whisker protraction.
A Optogenetic stimulation of Purkinje cells, using a 400 μm diameter optic fiber placed on the center of crus 1, induced whisker protraction at the end of the stimulus. Shown are the 100 trials of a representative experiment, split into the 50% of the trials with the largest and the 50% with the smallest protraction. **B** Heat maps of the coherence over time, showing predominantly activity in the theta band, that was not different between the groups of trials (**C**). N = 8 mice. Shades indicate SEM.

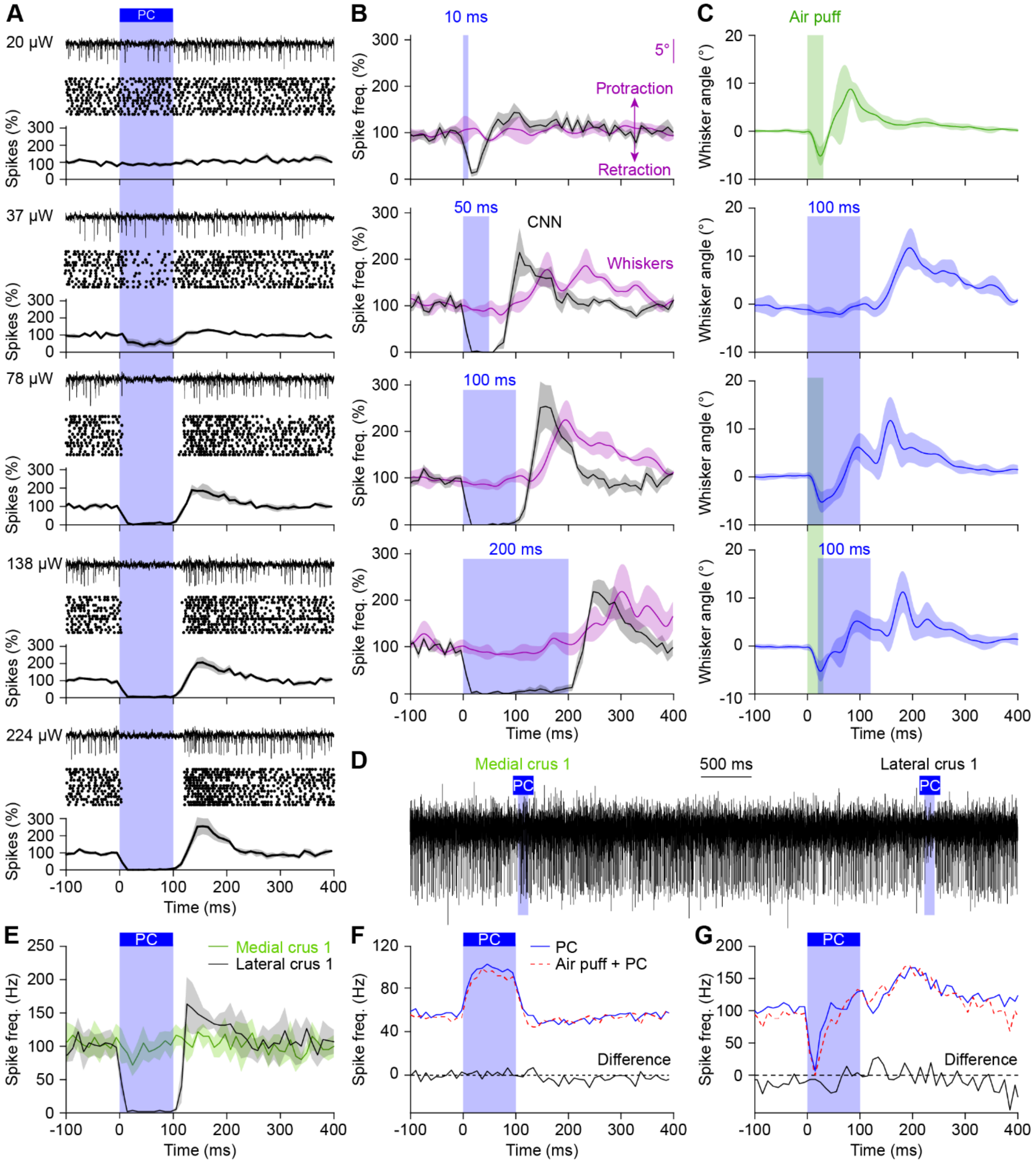

Fig. S20. Optogenetic stimulation of Purkinje cells silences neurons of the cerebellar nuclei.
A Using an optic fiber of 105 µm diameter and different illumination intensities, optogenetic stimulation of Purkinje cells induced a pause in firing of cerebellar nuclei neurons. At higher intensities, the pause was followed by rebound firing. For each intensity, we plotted an example trace, followed by a raster plot of the same experiment and the averaged peri-stimulus histogram of the spike rate (normalized to baseline) constructed from 6 responsive neurons in 2 mice. The shades indicate SEM. **B** Optogenetic stimulation could also trigger whisker protraction, but not during the period of stimulation itself. By varying the stimulus duration, we observed that the rebound firing in the cerebellar nucleus neurons (CNN) varied in timing and amplitude and that the whisker protraction followed the rebound firing. n = 6 cerebellar nucleus neurons in N = 2 mice. **C** Whisker air puff stimulation induced a reflexive protraction. This protraction was reduced

during the stimulus, but increased at the end of the stimulus. The whisker angle was normalized for each mouse at 0° before stimulus onset. N = 4 mice with each n = 100 trials per condition. Lines indicate average and shades SEM. **D** Extracellular recording of an exemplary cerebellar nucleus neuron displaying inhibition upon optogenetic stimulation of Purkinje cells in the lateral, but not the medial part of crus 1. For this experiment, an optic fiber with a diameter of 105 µm was used. **E** Averaged peri-stimulus histogram of two simultaneously recorded cerebellar nucleus neurons. The two neurons were separated laterally by 305 µm. **F** Exemplary recording of a Purkinje cell during whisker air puff stimulation alone and during combined air puff and Purkinje cell stimulation, revealing the absence of an additive impact of the sensory stimulation during Purkinje cell stimulation. **G** Example of a recording of a cerebellar nucleus neuron illustrating that in a subset of nucleus neurons, a transient, but complete block of output could be generated during optogenetic stimulation, even during trials during which also air puff stimulation to the whiskers was given.

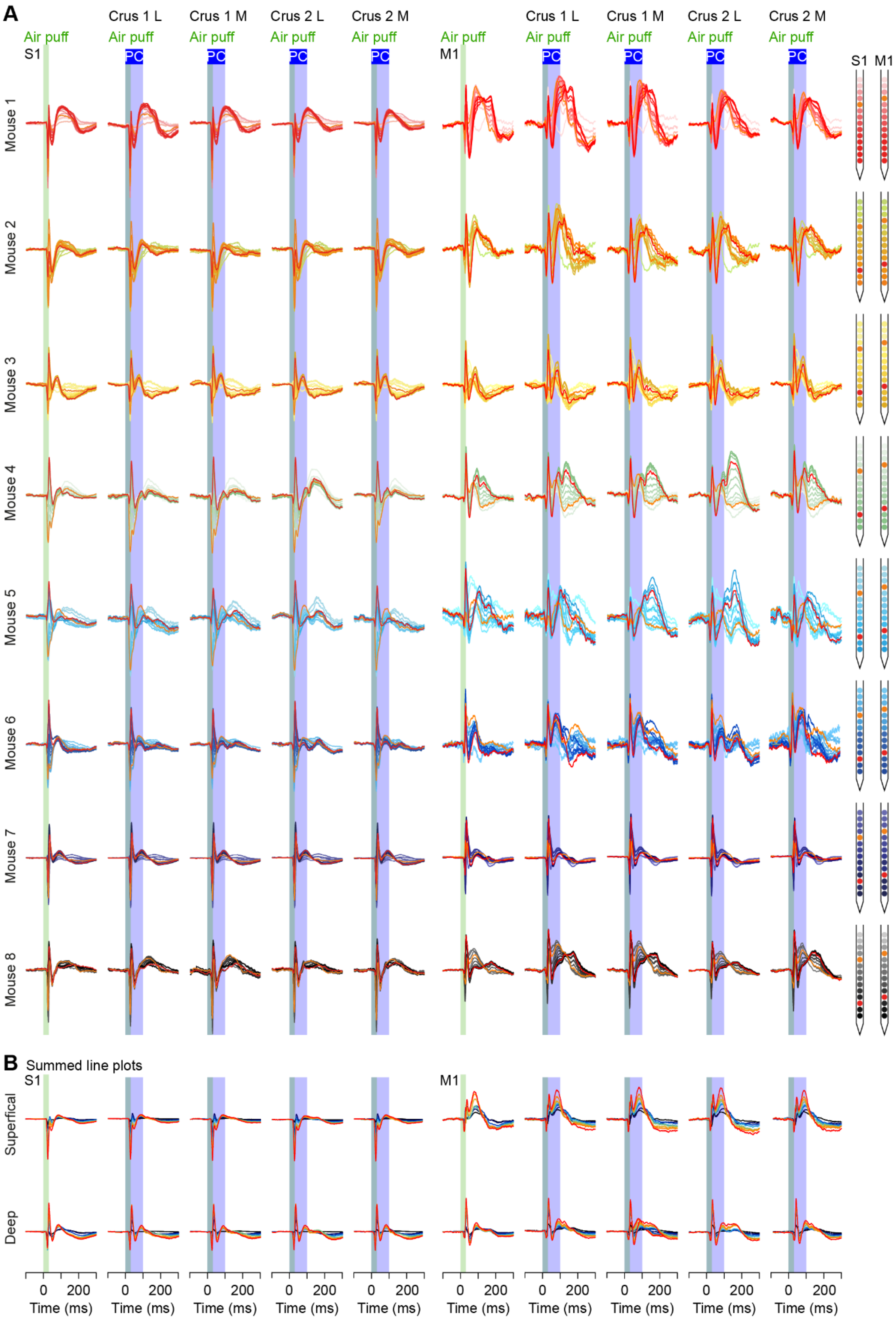
A
Crus 1 L
Crus 1 M
Crus 2 L
Crus 2 M
Air puff
PC
S1
M1
Mouse 1
Mouse 2
Mouse 3
Mouse 4
Mouse 5
Mouse 6
Mouse 7
Mouse 8
B Summed line plots
Superfical
Deep
0 200
Time (ms)

Figure S21. LFP recordings of individual mice share similar features.
A Each row shows the averaged LFP traces during the different stimulus conditions recorded in wS1 (left) and wM1 (right). Each color indicates a different electrode, with brighter colors corresponding to the more superficial and darker colors to the deeper channels, according to the schematics on the far right. Channels 3 and 10 are always indicated in orange and red, respectively. **B** Summed line plots of the channels 3 and 10 of each mouse normalized so that the red line indicates the average. Each color symbolizes one mouse, consistent with the color coding in **A**. This shows that all mice contributed to the different phases of the LPF responses. Air puffs were applied to the contralateral large facial whiskers and optogenetic stimulation was delivered with 105 µm optic fibers (delivering 0.2 mW) placed on the four locations indicated in Fig. S22A (inset). The label on top of the columns indicate which fiber was activated. L = lateral; M = medial.

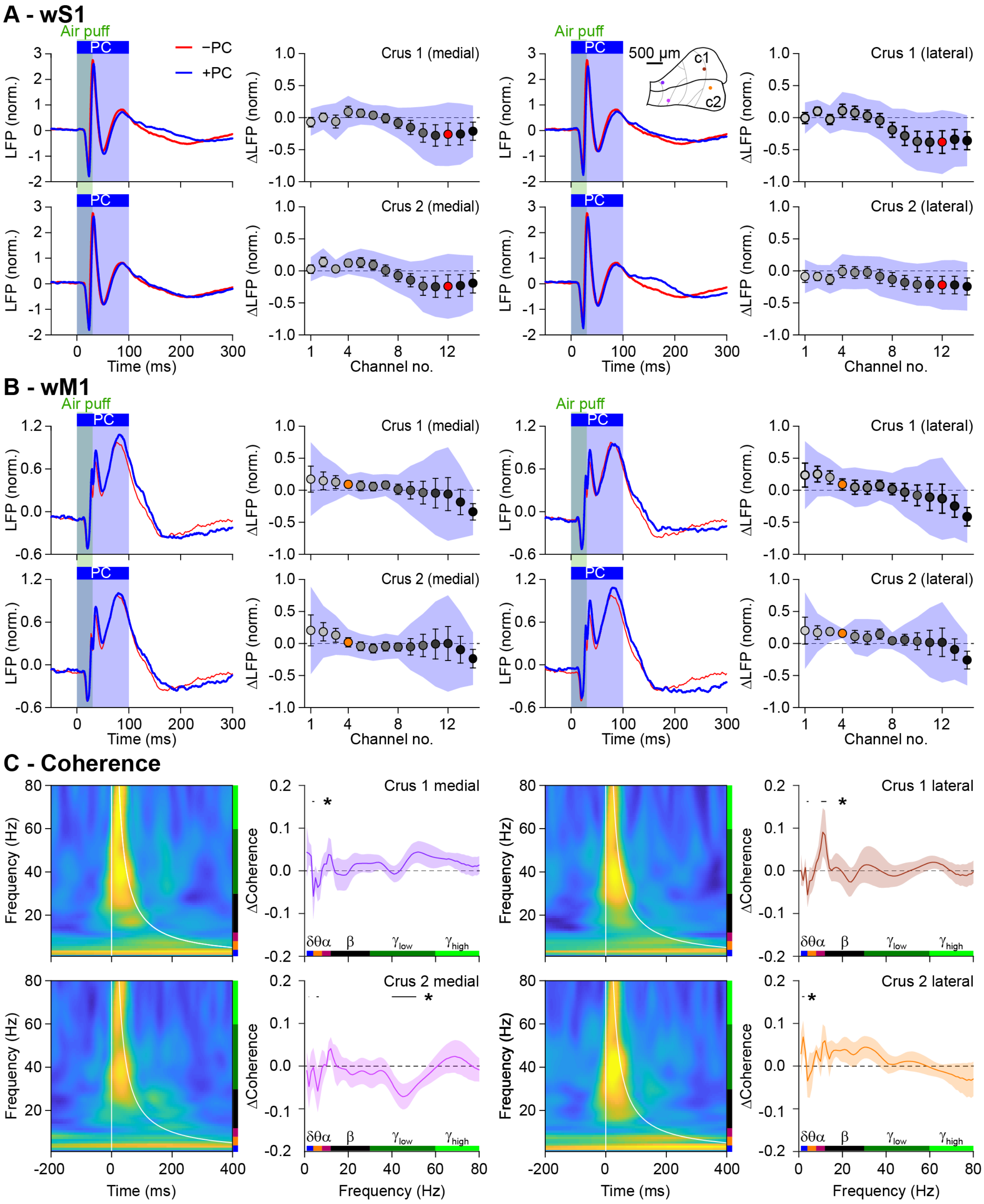

Fig. S22. Regional differences in the impact of Purkinje cell stimulation on sensory-induced local field potentials in wS1 and wM1.

A Averaged local field potentials (LFP) of the supra- (light colors) and subgranular (dark colors) of wS1 upon either only air puff stimulation of the contralateral facial whiskers (red colors) or air puff stimulation in combination with optogenetic stimulation of Purkinje cells (PC; blue colors). Optogenetic Purkinje cell stimulation was performed using four optic fibers with 105 µm diameter placed at different locations in crus

1 (c1) and crus 2 (c2; inset). During each trial, one of the fibers was activated in a random sequence. The 2nd and 4th columns indicate the difference in the amplitudes of the first positive peaks following stimulation, using the same color codes as in Fig. 1. Error bars indicate SEM and shaded areas sd. **B** The same analysis, but now for wM1. **C** Combined air puff whisker stimulation and optogenetic Purkinje cell stimulation resulted in gamma band coherence between wS1 and wM1 (heat maps). The coherence was different from those trials in which only the whiskers were stimulated, as indicated by the ΔCoherence plots. The impact of Purkinje cell stimulation on sensory-induced coherence depended on the location of optogenetic stimulation, with the lateral part of crus 1 and the medial part of crus 2 having opposite impact on gamma (but not theta) band coherence and the other locations having more intermediate effects. Lines are averages and shaded areas indicate SEM. *N* = 7 mice.

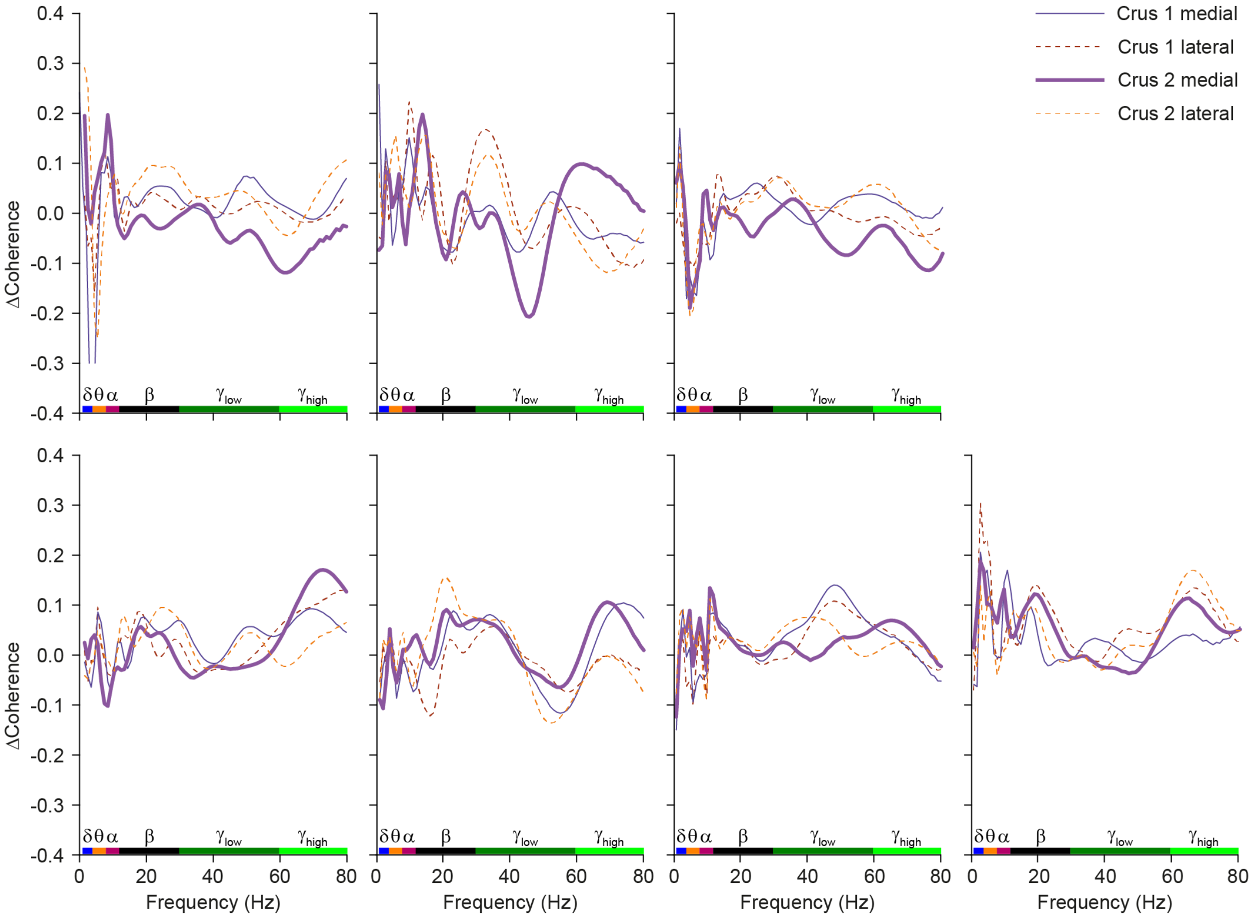

Fig. S23. Regional differences in the impact of Purkinje cell stimulation on wS1-wM1 coherence. Change in coherence comparing trials with only sensory stimulation and combined sensory and optogenetic Purkinje cell stimulation plotted separately for each of the 7 mice used to construct the averages displayed in SI Appendix, Fig. S22C. Each color indicates a different location of optogenetic stimulation.

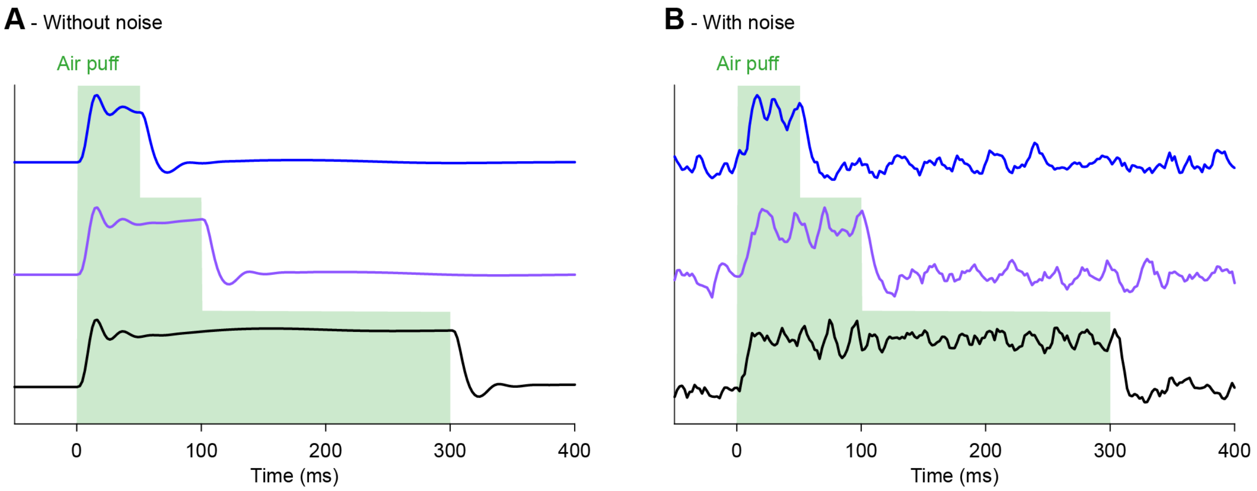

Fig. S24. Transient inputs can induce longer oscillations in the presence of noise.
A Modeled dynamics of the supragranular excitatory population of S1 in the presence of transient air puffs of different durations, and in the absence of internal noise. **B** Same simulation, but with the presence of a certain level of noise. The presence of noise allows for stochastic-driven oscillations beyond the first few cycles of the noiseless version. Connections to other layers and areas of the model, besides the connection to the local inhibitory population, have been removed to simplify the visualization of the dynamics. Local connectivity parameters as in Fig. 5.

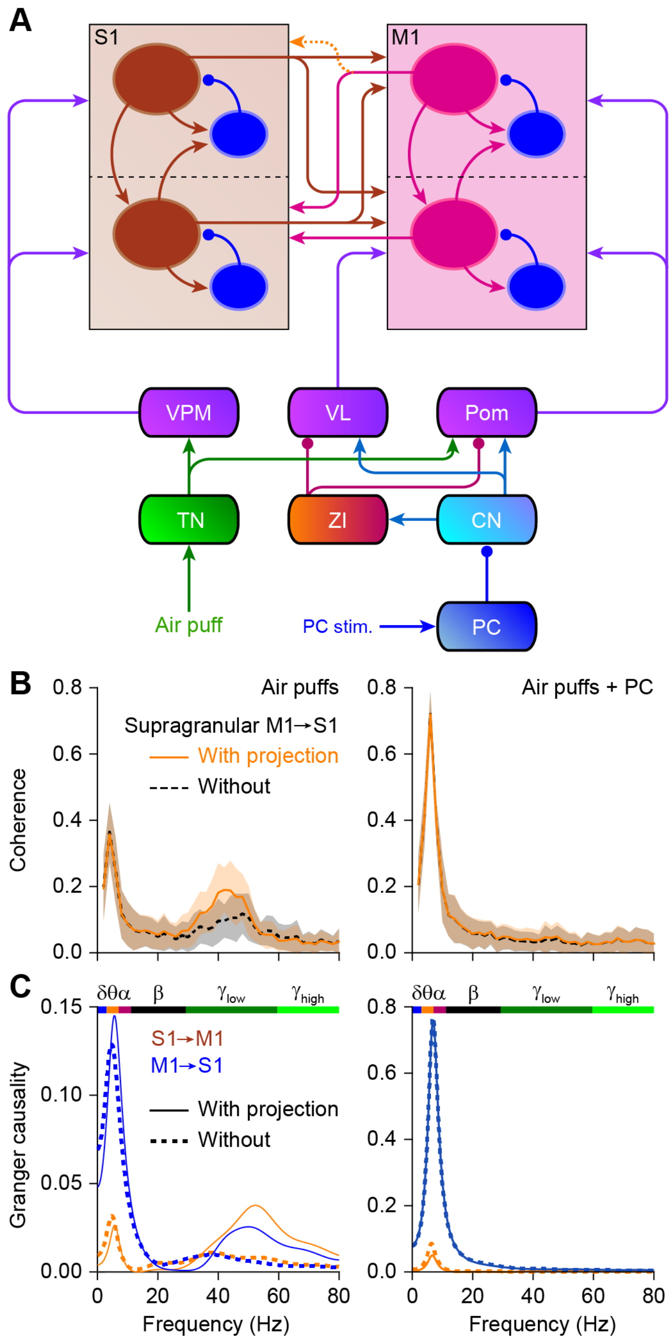

Fig. S25. Laminar model: impact of a projection for the supragranular layers of M1 to the supragranular layers of S1.

A Here we compared the circuit with and without a direct connection between the supragranular layers of M1 and S1 (dashed orange arrow). **B** Removing the supragranular M1 to S1 connection resulted in a slightly less powerful gamma band coherence (black) upon simulation of the trigeminal nuclei (simulating sensory input of the whiskers) than the same simulation in the presence of the supragranular M1 to S1 connection (orange). The impact of this connection was less during the combined trigeminal + Purkinje cell stimulation. C Granger causality analysis revealed that deleting the supragranular M1 to S1 connection resulted in a strong decrease of the contribution of both M1 and S1 to the sensory-induced gamma band coherence. Note that the situation with the supragranular M1 to S1 connection is the circuit that was used to generate the data of Fig. 5. These data are replicated here to facilitate comparison. Lines indicate averages and shaded areas sd.

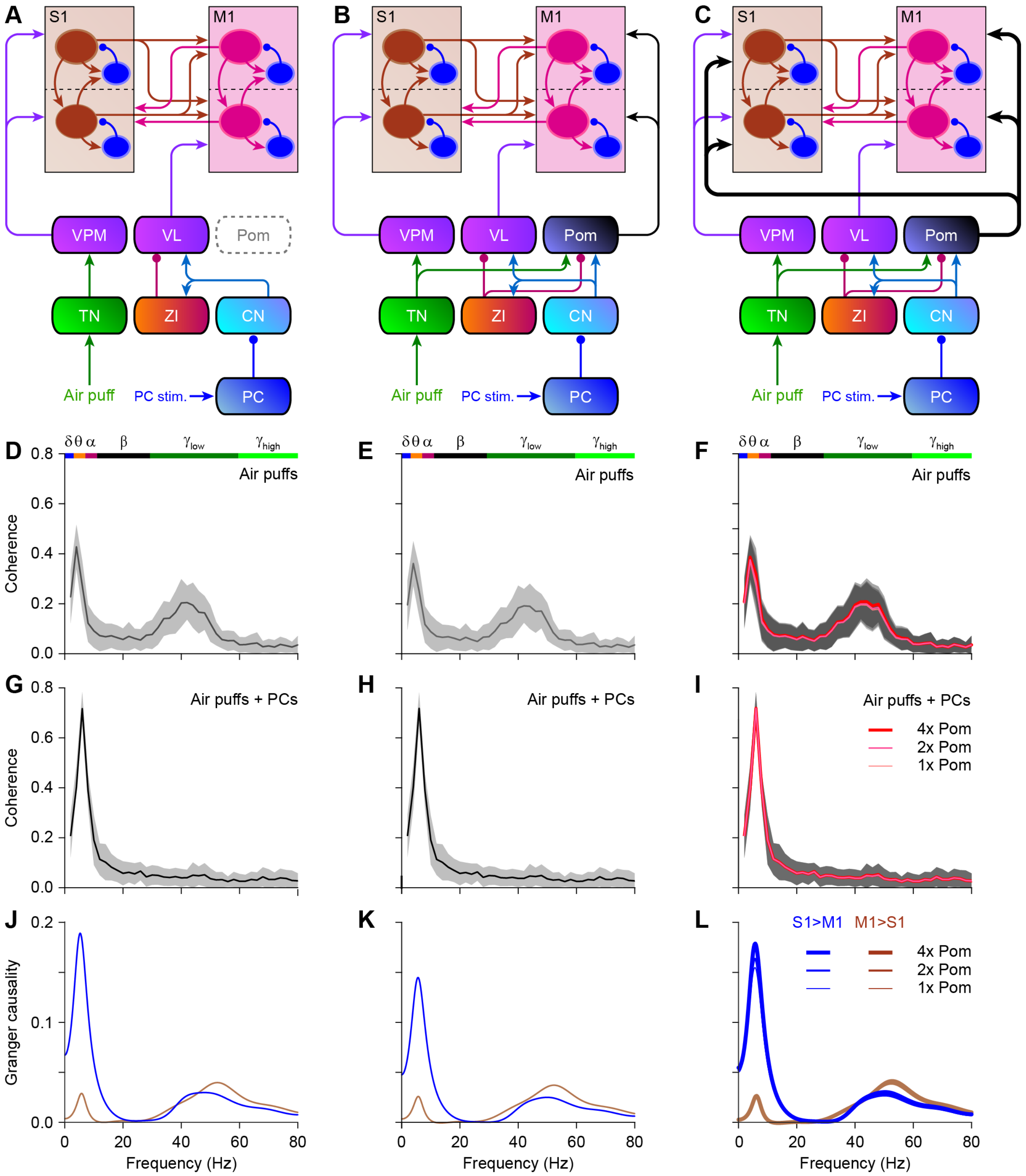

Fig. S26. Impact of Pom connectivity on laminar model

To test whether the Pom could affect the flow between S1 and M1 during gamma band coherence, we used our computation model in the configuration without a supragranular connection between M1 and S1 (Fig. S18). In this configuration, S1 is dominant over M1 when generating gamma band coherence. To study the impact of the Pom, we compared three different configurations: without Pom (**A**), with Pom projecting only to M1 (**B**) and with Pom projecting to both S1 and M1 with comparable strengths (**C**). Of the latter, we considered not only the normal connectivity strength from Pom to S1 (1x; see Methods), but also a value corresponding to two (x2) and four times (x4) this strength. **D-F** The different configurations did affect the amplitude of sensory-induced coherence between S1 and M1 (simulated by stimulation of the trigeminal

nuclei), but did not affect the frequency characteristics. **G**-**I** A similar observation was made for the conjunctive trigeminal + Purkinje cell stimulation. **J**-**L** Granger causality analysis demonstrated that the Pom could not induce M1 to be causative for sensory-induced gamma band coherence (as the direct supragranular connection between M1 and S1 could; see Fig. S25). Lines indicate averages and shaded areas sd.

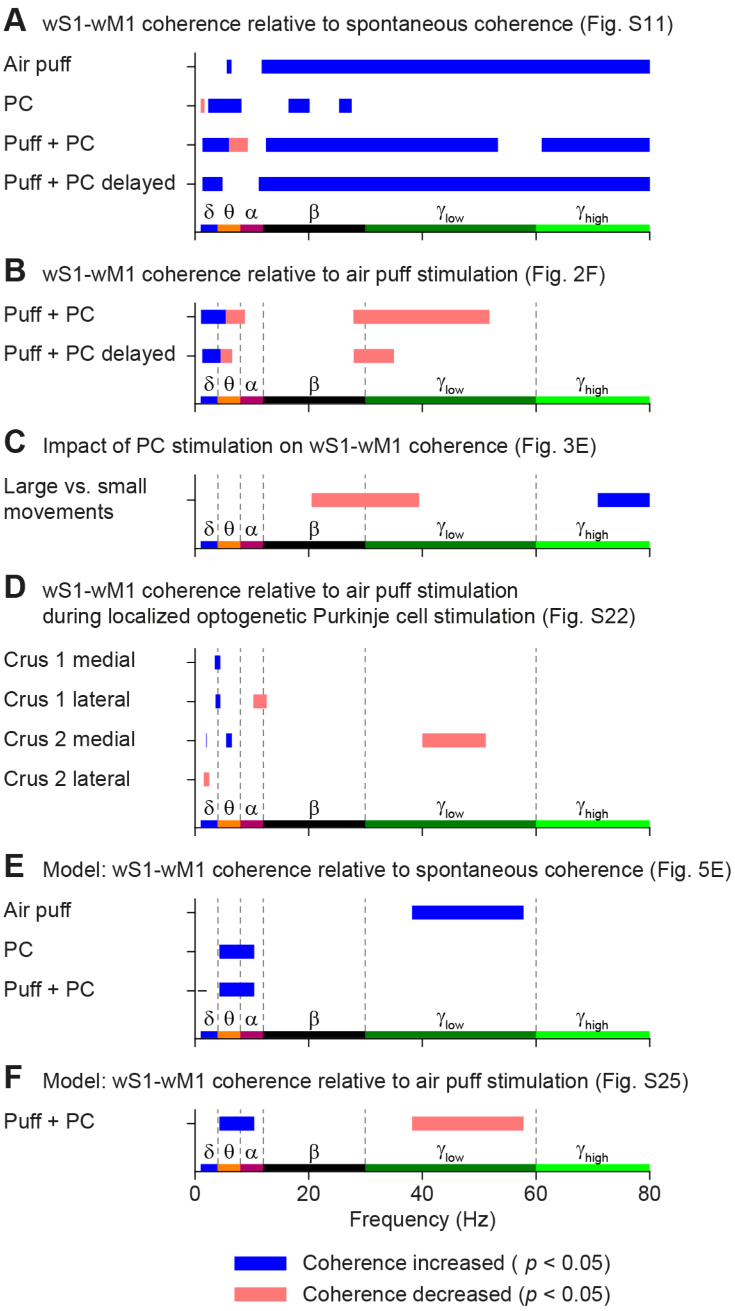

Fig. S27. Summary for interactions between different bands across areas and conditions. A-F Each panel exhibits the frequency intervals with significant interactions in wS1-wM1 coherence bands across conditions for all mice. For each panel a different source for the baseline is used. Colors indicate whether coherence significantly increases (blue) or decreases (red), relative to baseline coherence. **A** Relative to spontaneous coherence levels (Fig. S11), the majority of stimulation conditions leads to an increase of coherence. The single air puff stimulus leads to increased coherence across the higher frequency bands. Stimulation of PCs leads to a significant decrease in coherence across the spectrum, with exception of interactions between the lower bands. **B** Relative to air puff stimulation, PC+Puff stimulus leads to a clear decrease in the low gamma range, which is less prevalent when the PC stimulation is delayed. **C** For larger whisker movements coherence is significantly increased in the high gamma band, but decreased between beta and low gamma bands. **D** Confirming previous findings under pharmacological manipulations the largest decrease of coherence is observed in the gamma band when the medial region of crus 2 is stimulated. **E** The model shows an increase in lower bands for PC stimulation and higher bands for Air puff stimulation. **F** The model also shows decrease of coherence during stimulation in gamma band and an increase in the lower part of the spectrum.

	N	*p*	Test statistic	Sign.?	Test
**First negative peak (Fig. S6B)**<br>*Air puff vs. Air puff + simultaneous PC stimulation*					
wS1 [supragranular layers]	8	0.742	W = -6.0	no	Wilcoxon matched-pairs
wS1 [layer IV]	8	0.195	W = 20.0	no	Wilcoxon matched-pairs
wS1 [subgranular layers]	8	0.148	W = 22.0	no	Wilcoxon matched-pairs
**First positive peak (Fig. 1)**<br>*ANOVA on the three trial conditions*					
wS1 [supragranular layers]	8	0.607	$\chi^2(1) = 1.000$	no	Friedman's ANOVA
wS1 [layer IV]	8	0.325	$\chi^2(1) = 2.250$	no	Friedman's ANOVA
wS1 [subgranular layers]	8	**0.008**	$\chi^2(1) = 9.750$	Yes	Friedman's ANOVA
Air puff vs. Air puff + simultaneous PC stimulation		***0.024***	*2.250*	*yes*	*Dunn's post-hoc*
Air puff vs. Air puff + delayed PC stimulation		*0.453*	*-3.000*	*no*	*Dunn's post-hoc*
Simultaneous vs. delayed stimulation		***0.003***	*-0.750*	*yes*	*Dunn's post-hoc*
wM1 [subgranular layers]	8	**0.030**	7.000		Friedman's ANOVA
Air puff vs. Air puff + simultaneous PC stimulation		0.617	-0.500	*no*	*Dunn's*
Air puff vs. Air puff + delayed PC stimulation		**0.012**	-2.500	*yes*	*Dunn's*
Simultaneous vs. delayed stimulation		0.046	-2.000	*no*	*Dunn's*
wM1 [subgranular layers]	8	0.008	9.750		Friedman's ANOVA
Air puff vs. Air puff + simultaneous PC stimulation		***0.024***	*2.250*	*no*	*Dunn's*
Air puff vs. Air puff + delayed PC stimulation		*0.453*	*-3.000*	*yes*	*Dunn's*
Simultaneous vs. delayed stimulation		***0.003***	*-0.750*	*yes*	*Dunn's*

Table S1. Statistical evaluation of the data represented in Figs. 1 and S6.
For each mouse (*N* = 8), 100 trials for each condition were recorded and averaged. For each channel, the first minimum of the LFP signal (see red arrow in Fig. S6A) and the first maximum (see black arrows in Fig. 1C-D) were calculated and averaged for channels 2-5 (supragranular layers) and channels 10-13 (subgranular layers). The granular layer (layer IV in wS1) was represented by channel 8. Trial types were presented in a pseudo-random order. We compared the amplitude of the first negative LPF peak after air puff whisker stimulation (see red arrow in Fig. S5A) between trials with only air puff and combined air puff and optogenetic Purkinje cell stimulation. Next, we compared the amplitude of the first positive LFP peak (see e.g. the black arrows in Fig. 1C-D). As this peak was affected, we compared here three trial types: air puff, air puff + simultaneous Purkinje cell stimulation, and air puff + 20 ms delayed Purkinje cell stimulation. The analysis was done for the three layers separately, and significance is noted after Benjamini-Hochberg correction (yes or no in column "Sign.?"). Because an effect was observed, subsequent post-hoc tests were done and these were again subjected to Benjamini-Hochberg correction for multiple testing. Note that wM1 was tested only for the positive peak, as the initial negative peak was very small.

SI References

1. R. Oostenveld, P. Fries, E. Maris, J. M. Schoffelen, FieldTrip: Open source software for advanced analysis of MEG, EEG, and invasive electrophysiological data. *Comput Intell Neurosci* **2011**, 156869 (2011).
2. T. P. Zanos, P. J. Mineault, C. C. Pack, Removal of spurious correlations between spikes and local field potentials. *J Neurophysiol* **105**, 474-486 (2011).
3. A. M. Amjad, D. M. Halliday, J. R. Rosenberg, B. A. Conway, An extended difference of coherence test for comparing and combining several independent coherence estimates: theory and application to the study of motor units and physiological tremor. *J Neurosci Methods* **73**, 69-79 (1997).
4. A. Borst, F. E. Theunissen, Information theory and neural coding. *Nat Neurosci* **2**, 947-957 (1999).
5. D. Chicharro, On the spectral formulation of Granger causality. *Biological cybernetics* **105**, 331-347 (2011).
6. T. Schreiber, Measuring information transfer. *Phys Rev Lett* **85**, 461-464 (2000).
7. M. Kaminski, M. Ding, W. A. Truccolo, S. L. Bressler, Evaluating causal relations in neural systems: granger causality, directed transfer function and statistical assessment of significance. *Biological cybernetics* **85**, 145-157 (2001).
8. I. Perkon, A. Kosir, P. M. Itskov, J. Tasic, M. E. Diamond, Unsupervised quantification of whisking and head movement in freely moving rodents. *J Neurophysiol* **105**, 1950-1962 (2011).
9. N. Rahmati *et al.*, Cerebellar potentiation and learning a whisker-based object localization task with a time response window. *J Neurosci* **34**, 1949-1962 (2014).
10. V. Romano *et al.*, Functional convergence of autonomic and sensorimotor processing in the lateral cerebellum. *Cell Rep* **32**, 107867 (2020).
11. L. Kros *et al.*, Cerebellar output controls generalized spike-and-wave discharge occurrence. *Ann Neurol* **77**, 1027-1049 (2015).
12. L. W. J. Bosman *et al.*, Anatomical pathways involved in generating and sensing rhythmic whisker movements. *Front Integr Neurosci* **5**, 53 (2011).
